# Achieving Harmony among Different Social Identities within the Self-Concept: The Consequences of Internalising a Group-Based Philosophy of Life

**DOI:** 10.1371/journal.pone.0137879

**Published:** 2015-11-30

**Authors:** Felicity M. Turner-Zwinkels, Tom Postmes, Martijn van Zomeren

**Affiliations:** Department of Social Psychology, University of Groningen, Grote Kruisstraat 2/1, 9712 TS, Groningen, The Netherlands; University of New South Wales, AUSTRALIA

## Abstract

It can be hard for individuals to manage multiple group identities within their self-concept (e.g., being a Christian and a woman). We examine how the inter-identity fit between potentially conflicting identities can become more harmonious through a self-defining group philosophy for life. Specifically, we test the hypothesis that *holistic* group identities (based in group philosophies for life that prescribe the behavior of their members in any situation, such as religion) become more strongly related to other identities in the self-concept (e.g., gender) when they are strongly *self-defining* (i.e., devotedly applied to daily life). In three studies we investigated the inter-identity fit between individuals’ (highly holistic) religious identity and (less holistic) gender identity. Results provided converging support for our hypothesis across diverging methods (explicit questionnaires, more implicit associations, and a novel network analysis of group traits). We discuss the importance of understanding how some (i.e., holistic and self-defining) group identities may harmonize otherwise less harmonious group identities within one’s self-concept.

## Introduction

Although much is known about different identities that comprise the self (e.g., [1]), less is known about how and why these identities fit together within the larger self-concept. This is important to understand because *inter-identity fit* (i.e., an individual’s perception of the degree of overlap between multiple identities within the self-concept) has significant consequences for personal well-being (e.g., coping, self-esteem and stress; [2–4]) and behaviour towards others (e.g., discrimination; [5]). For example, research suggests that individuals’ well-being during the life-transition to university was higher when they felt that their ‘old’ high-school identity was compatible with their ‘new’ university identity [6]. In fact, research has documented evidence for many types of inter-identity fit [7, 8], ranging from negative [9], separateness or independence [10], to strongly overlapping [11, 12]. Despite the importance of understanding how multiple identities fit together within the self-concept, however, we still know very little about what promotes such fit.

We focus on two factors that could promote inter-identity fit: (a) the capacity of a group identity to provide a philosophy of life, and (b) the extent to which this philosophy is personally put into practice, or applied, by an individual. The first factor we thus investigate is the group characteristic of *holisticness*, which we define as the capacity for a group to provide a philosophy for life for people in general. We propose that groups are perceived to differ in this capacity: Groups that provide a strong philosophy for life try to determine behavior *across all* situations; Groups that do not provide a strong philosophy but simply prescribe what is normative and anti-normative in *specific* situations (e.g., on a football pitch, in church). Holisticness can have important consequences for the inter-identity fit among different social identities within the self-concept. When one of those social identities is derived from a more holistic group which provides a strong philosophy for life (i.e., norms that prescriptively transcend specific situations; e.g., group members should *always* be caring), then it is likely that this will also change an individual’s behavior in contexts within which *other* social identities (e.g., gender, work, or sports) would otherwise be dominant. A result of this would be that holistic identities would acquire stronger inter-identity fit (i.e., overlap with other social identities). A prototypical example of a group high in holisticness is religion (e.g., Christianity, Islam, Buddhism) because it both seeks to [13] and can achieve wider influence on its members’ lives [14, 15].

Although a holistic group provides a philosophy for life that promotes inter-identity fit, we propose that what ultimately determines this fit is the way it is applied by individuals. This brings us to the second factor under study in the present paper: the individual characteristic of *self-definingness*, which we define as the extent to which an individual actually puts into practice a group’s philosophy to guide their life in general (for a discussion on the distinction between identification and self-definingness see “theoretical notes” in S1 Appendix). When holistic group philosophies are devotedly applied by an individual to their life in general (i.e., in a way that transcends specific situations), it is self-defining, providing the individual with meaning in life (e.g., a devoted Christian who applies Christian ideologies at work, home with friends, etc.). Thus, self-definingness concerns the extent an individual uses a group’s philosophies across all situations.

We expect that stronger inter-identity fit between multiple identities is an outcome of an individuals’ devoted application of holistic group philosophies (i.e., using to them across one’s life). For instance, when members of a holistic group that prescribes ‘caring’ behaviour experience this group as self-defining, they will aspire to uphold this norm throughout all areas of life. This will cause the trait ‘caring’ to be associated not just with the holistic group, but also with various other social identities such as gender or work. More technically, self-definingness will lead to stronger inter-identity fit in holistic groups, but less so in groups that are not holistic. We note that this active explanation best characterises strong self-definingness (e.g., religious individuals who use group philosophies in all areas of their life; e.g., [16]). Weak self-definingness, in contrast, concerns a reduction in or lack of effort to actively apply group philosophies.

To examine this idea, we focus on two specific social identities: religious identity (assumed to be relatively high in holisticness) and gender identity (assumed to be relatively low in holisticness; these assumptions will be examined in a pilot study). With this focus in place, we examined our hypothesis in three different studies that employed different methods to measure inter-identity fit (i.e., an individual’s perception of the degree to which multiple identities within the self-concept overlap). This set of studies contributes to the literature in at least two important ways: First, we advance theoretical knowledge of the factors that promote inter-identity fit amongst multiple identities within the self-concept. Second, we develop and use new methods that allow us to probe deeper into social identity content, which greatly facilitates the study of inter-identity fit.

### Positive (or negative or no) interdependence among identities?

Scholars in the social identity tradition (e.g., [17, 18]) originally assumed that a negative relation existed between different aspects of identity within the wider self-concept [19, 20]. According to this perspective, as personal identity (i.e., the unique part of the self which concerns distinctiveness from others) becomes salient, individuals become less able to perceive the self in terms of its group membership(s) and similarities with others (social identity; i.e., the part of the self that is socially shared with others, including one’s group memberships; [17, 21–23]). Multiple social identities also ‘compete’ with one another, in order to become the “focal category” (e.g., metacontrast; [23], p.455). This means that, at least at times, multiple social identities can inhibit each other. For example, the more an individual acts in terms of their lawyer identity, the less they act in terms of their maternal female identity [9, 24, 25]. Thus, although it is generally recognised that personal and (multiple) social aspects of the self are not completely independent, they are often researched as if they were mutually exclusive [19, 26].

This theoretical background may be one reason why the question of inter-identity fit (i.e., degree of multiple identities overlap) has received little attention. However, more recent research implies that identities need not always be negatively related (see [27], for an overview). For instance, there are specific conditions (e.g., when group status or identity continuity is high) in which inter-identity fit becomes more strongly overlapping [28–30]. Moreover, studies that consider inter-identity fit (e.g., [31–33]) demonstrate that subordinate identities can be successfully embedded in a superordinate entity. Similarly, research on crossed categorizations suggests that two categories can be successfully integrated within a superordinate identity (“we”) as long as the two subcategories are not considered highly self-relevant [34]. Thus, there are several conditions under which hierarchically structured social identities are somewhat overlapping.

A growing body of research acknowledges that inter-identity fit can exist between potentially orthogonal identities as well (cf. the notion of “intersectionality” see [35, 36]). For example, Roccas and Brewer’s [37] work on social identity complexity presents four key types of inter-identity fit, ranging from compartmentalised (or independent) to more interdependent (e.g., dominance) relations. Research has, however, not extended substantially beyond these informative, yet predominantly descriptive studies (e.g., describing the management of fit between two social identities; [38, 39]). The cognitive-developmental model of social identity integration [40] is one notable exception. In this model the authors discuss how inter-identity fit can develop because of individual-level antecedents such as personal coping and perceived social support, which facilitate potentially effortful integrative processes that lead to stronger inter-identity fit (e.g., [11, 38]). However, in the current paper, we argue that this analysis overlooks the importance of the *type* of identity being managed (e.g., holisticness), which is a group characteristic.

### Holistic groups and self-defining identities

Baray et al. [41] approached the question of how multiple identities fit together by focusing on a ‘special’ type of group with particular normative content (for an alternative identity fusion account, see [42]). Groups that prescribe a philosophy for life (e.g., a religious group) can paradoxically strengthen the personal identity of individuals who apply that philosophy (e.g., make an individual more certain of their personal attitudes or convictions). For example, an individual may use the group’s philosophy (e.g., Biblical teachings) to guide their individual behaviour in everyday life (e.g., motivating a personal decision about whether to prioritize self-achievement or self-enhancement goals), endowing them with a sense of certainty and self-assuredness about what is important to them personally. As a consequence, these social identities derived from highly holistic groups can promote a fit between that specific *social* identity and an individual’s *personal* identity [41, 43]. In contrast, groups that do not provide such a normative recipe for life only influence an individual in situ (e.g., TV show followers). Therefore, these groups low in holisticness only inform a *specific* social identity and this remains independent from other identities that an individual may possess.

Importantly however, Baray et al. [41, 43] did not study the inter-identity fit between different social identities. We move beyond this theoretical framework by exploring the inter-identity fit among multiple social identities. We focus on two specific social identities selected on the basis of a pilot study: religious (relatively high in holisticness) and gender identity (relatively low in holisticness). Specifically, we will investigate to what extent this holistic religious identity becomes more strongly fitting with an otherwise potentially non-overlapping or even conflicting social identity, in this case gender. In this way, we advance our understanding regarding the organisation of the self-concept by thoroughly exploring the inter-identity fit between two specific identities.

We further build on Baray et al.’s research [43] in two key ways. Firstly, we suggest that groups vary on a continuum from more holistic to less holistic (cf. Baray et al. [41], who relied on a dichotomy of self-defining vs. non self-defining). Secondly, we suggest that is it important differentiate holisticness—a *group characteristic* concerning the capacity for a group to prescribe a philosophy for life, which is socially shared and exists to some extent outside of the self—from the *individual characteristic* of the personal application of group philosophies to life in general. Notably, where we now refer to groups as more or less holistic, Baray et al. [41] used the term self-defining. We changed this terminology because we wanted to clearly differentiate the holisticness of groups—a group characteristic—from the subjectively applied self-definingness of social identities—a personal characteristic. Unlike Baray et al. [41], we propose that the extent to which an individual applies holistic group philosophies (i.e., self-definingness) will determine how strongly the social identity fits with other identities. Even though the group may have the capacity to be holistic (i.e., it prescribes a philosophy for life), individuals vary in the degree to which this identity is self-defining.

For instance, some may act in ways consistent with their religious identity only when in church, others in more or less every situation (cf. [14]). For the former, religion is less self-defining than for the latter. The implication is that a holistic based identity that is only weakly self-defining remains relatively independent from other identities (i.e., Christian identity is active at church, administrator identity is active at work). In contrast, a strongly self-defining holistic based identity increases inter-identity fit with ancillary social identities, which might otherwise be in conflict with it (i.e., Christian identity being active at church, and at work). Notably, the influence of group philosophies should be visible not only in group members (e.g., devoted Christians) but also those who do not consider themselves group members but still share and apply some elements of the group’s values (e.g., use of Christian values by people who grew up in a Christian culture). Indeed, what is ultimately important in promoting inter-identity fit is the personal application of holistic group philosophies across situations (i.e., to one’s life).

### Overview

We proposed that holistic groups have the capacity to provide a philosophy for life that can promote inter-identity fit within the self-concept, provided the individual considers it strongly self-defining (in the sense that they actively apply the group’s philosophies to guide their life). Thus, holisticness is defined as a group characteristic (i.e., how is the group seen in general) and self-definingness as an individual characteristic (i.e., how do you personally experience the identity). To examine this proposal, three studies investigate the inter-identity fit (i.e., an individual’s perception of the degree to which multiple identities within the self-concept overlap) between multiple (religious [more holistic] and gender [less holistic]) social identities as a function of their self-definingness. Our central hypothesis is that whether self-definingness promotes inter-identity fit depends on holisticness: When religion (as a holistic group) is self-defining, it will promote inter-identity fit and will therefore be strongly overlapping with gender identity. Conversely, when gender (as a less holistic group) is self-defining, it will not promote inter-identity fit and will therefore be non-overlapping with religious identity. (For a more detailed discussion about expectations concerning groups low in holisticness, see S2 Appendix.)

Importantly, we used different methodologies to tap into the notion of inter-identity fit between religion and gender. Study 1 focused on self-reported perceptions of inter-identity fit (e.g., whether the values of one social identity overlap with another). Study 2 more directly taps into the underlying cognitive representation of inter-identity fit by using relatively implicit measures of response times to traits associated with religion and gender to assess degree of cognitive overlap among multiple social identities (applying an experimental paradigm adapted from Aron, Aron, Tudor and Nelson [44], and Smith and Henry [45]). Finally, in Study 3 we developed a new measure of social identity content to assess inter-identity fit (i.e., a semantic network analysis of associated traits and characteristics which assesses the fit and overlap of actual identity content). Through the use of a multi-method approach assessing inter-identity fit, we seek convergent evidence for our core proposal that holistic groups promote inter-identity fit when individuals apply group philosophies to guide their life in general.

## Pilot

The Pilot Study had two key objectives: First, to select two groups which differ in holisticness to focus on in subsequent studies; second to test our hypothesis that the consequences of self-definingness for the strength of inter-identity fit is moderated by holisticness. Five groups were selected for study; Christianity, Environmental groups, Healthy-living groups, Gender and Nationality. These groups were selected on *a priori* grounds because we expected them (a) to differ in holisticness, and (b) to prescribe content (e.g., philosophies) which are reasonably consistent or consensually shared across group members. So, although other groups—such as family or work—are also likely to be high in holisticness, the content of their ideologies is also likely to differ strongly according to one’s specific role (e.g., a builder would be expected to have very different content from a social scientist), which would strongly limit the assessment of holisticness. We hypothesized that the first three groups (Christianity, Environmental and Healthy-living) would be perceived as prescribing a philosophy for life (i.e., highly holistic). It was expected that the more strongly self-defining these social identities were, the more strongly they would fit or overlap with other social identities within the self-concept. In contrast, we expected the latter two groups (Gender and Nationality) to be perceived as less holistic and therefore unrelated to inter-identity fit.

### Method

#### Sample

One-hundred-and-thirty-three participants were recruited online via the US internet survey site Mechanical Turk, participating in exchange for ¢60 [46]. Fifteen participants were excluded due to substantially incomplete answers. A further 4 participants were removed because they were of a non-Christian faith (e.g., Jewish, Universalist, and Islamic). Thus, individuals who would view Christians as an outgroup were excluded. Those remaining were not anti-religion, and especially given the US cultural context, we expected aspects of Christian heritage to inform their identity (e.g., [47]), thus allowing us to explore the consequences of the use of the group’s holistic philosophies rather than category membership. The final sample of 114 participants consisted of 32 Males and 82 females, aged between 19 and 69 years (M = 32.99, SD = 12.47). Of this, 48 classified themselves as religious, and identified with a denomination of Christianity. A further 57 classified themselves as non-religious and 8 as unsure, however 19 of these participants continued to identify with a denomination of Christianity, confirming our assumption that although non-religious, many individuals identify with aspects of Christian heritage (the remaining participant was unspecified).

#### Ethics statement

Written consent was obtained from all subjects, and all studies reported in this paper were approved by the “Ethical Committee Psychology”, at the University of Groningen.

#### Procedure

Participants completed the study—entitled “Group memberships”—individually over the internet. Before electing to participate, participants read a short study description explaining that they would be asked to answer questions about some of the different groups they belong to. Participants then followed a link on screen to start.

Participants were asked to answer a set of questions about each of the five groups, in the following order; Gender, Nationality, Christianity, Healthy-living, Environmental. All items were scored on 7-point Likert-type scales (1 = strongly disagree, 7 = strongly agree).

Three new scales were developed in order to operationalize the extent to which a social identity was considered to be holistic, self-defining, and to fit with multiple social identities. Although a more extensive set of items was developed for each of these three scales (see Study 1 and S3 Appendix), a limited set was used in the Pilot due to the lengthy and repetitive nature of answering the same questions for five different groups.

*Holisticness* of social identity was comprised of two items which aimed to assess the extent individuals perceived a group as having the capacity to define all aspects of life for people in general ([group name] provides a philosophy for life, [group name] teaches people how to live, *r* = .58—*r* = .91). *Self-definingness* used three items to measure the extent that an individual considers their life in general to actually be guided by the philosophies provided by each of the five groups: ([group name] helps me see what is important in life, [group name] gives me a sense of purpose, [group name] steers me through life; α = .84—α = .99). (For a factor analysis exploring the distinction between the self-definingness and holisticness scales, see S4 Appendix.)

Several (four or six) items comprising the *inter-identity fit* scale were used to measure an individual’s explicit representation of the overlap between multiple social identities. These items assessed the fit of values and group identity content (The values of [group name 1] are compatible with the values of [group name 2], Being a member of [group name 1] is consistent with being a member of [group name 2]). Inter-identity fit was not measured between all groups (i.e., 5x5), but only those of differing (anticipated) holisticness. So, the fit of each of the three groups expected to be more holistic (i.e., Christian, Healthy-living, and Environmental) was measured with the two remaining, less holistic groups (i.e., Gender and Nationality; resulting in a four item scale), and vice versa (resulting in a six item scale). These items were then averaged to find the overall association of that specific identity with multiple social identities (*α* = .86—*α* = .89).

*Group identification* was measured in order to assess one’s relation with the group using a modified version of Doosje, Ellemers and Spears’ [48] four-item scale (e.g., I see myself as a [group name], see [49], p. 615 for scale details). As before, identification was measured with all five groups (*α* = .78–*α* = .98).

Finally, demographic variables including age, gender, nationality and religion were measured. Participants were also asked whether they were religious (trichotomous response; yes, no, unsure). Participants were then thanked and debriefed.

### Results

**Fig 1:**
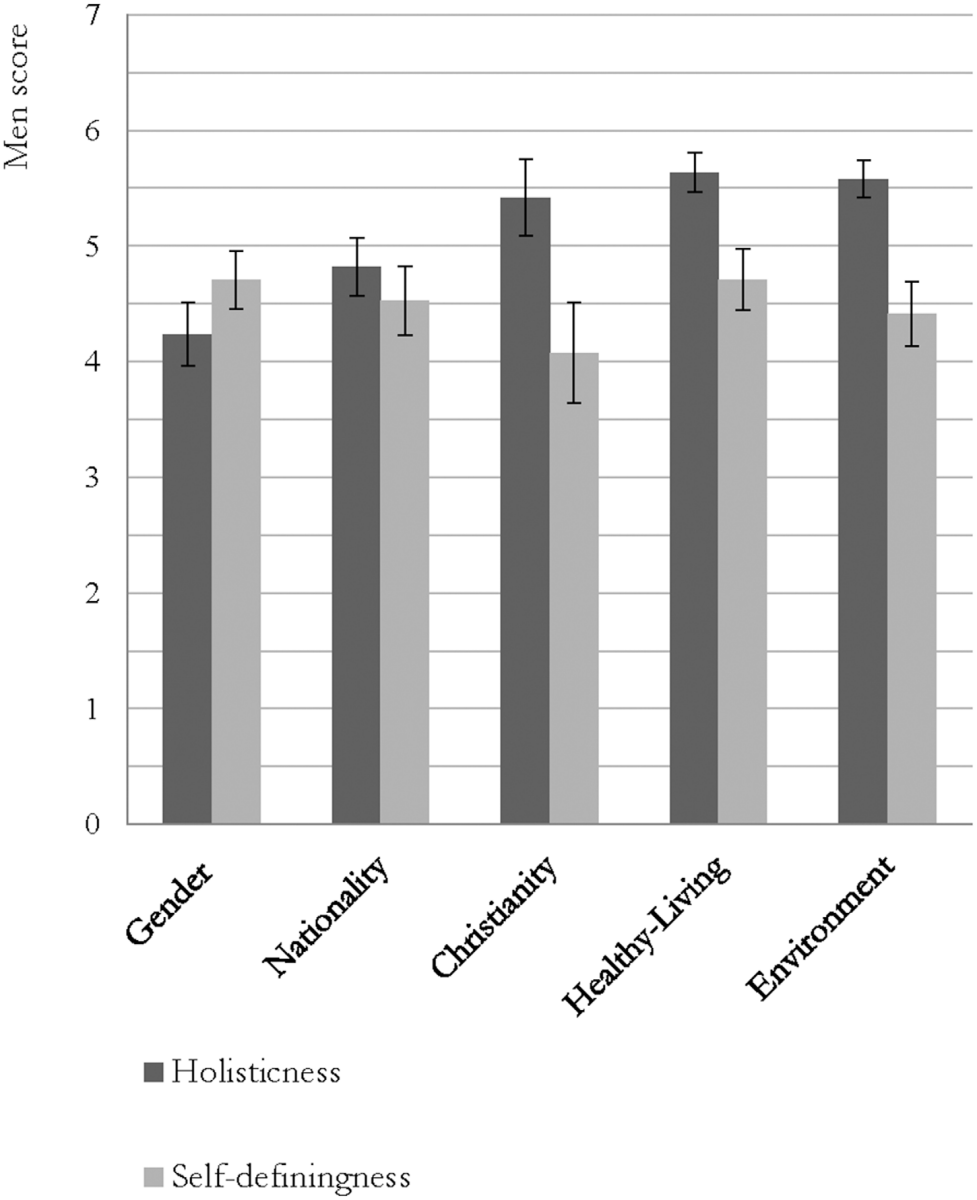
Mean perceived holisticness and self-definingness of Gender, Nationality, Christianity, Healthy-Living and Environment.

In order to select the most suitable identities for further investigation in Studies 1–3, holisticness and self-definingness means were inspected for each group (see Fig 1). A repeated measures analysis of variance (RM-ANOVA; with Greenhouse-Geisser degrees of freedom due to a sphericity violation; *W* (9) = .43, *p <* .001) showed that Gender and Nationality were perceived to be less holistic than Christianity, Healthy-living and Environmental groups (*F* (2.96, 334.79) = 27.03, *p* < .000001, *η*_*p*_^2^ = .19). Bonferroni corrected pairwise contrasts confirmed that Gender and Nationality were significantly less holistic than all three other groups. Furthermore, Nationality occupied an intermediate position, in that it also differed significantly from Gender. RM-ANOVAs (with Greenhouse-Geisser degrees of freedom due to a sphericity violation; *W* (9) = .46, *p <* .001) on self-definingness revealed some significant differences between groups (*F* (2.79, 315.66) = 3.68, *p* < .01, *η*_*p*_^2^ = .03), although effects were much less strong. Bonferroni corrected pairwise contrasts revealed that Gender and Healthy-living groups were experienced as significantly more self-defining than Christianity, but no further differences were found.

Thus, the pilot data largely confirmed expectations: Gender scored low on holisticness; Christianity, Healthy-living and Environmental groups were high in holisticness. Nationality emerged with higher holisticness than expected. In contrast, groups did not tend to differ so substantively in self-definingness (which emphasizes the utility of this variable in our planned moderation analysis). For this reason, we also tested which identities would have most potential to affect inter-identity fit. We thus tested our hypothesis that the impact of self-definingness for inter-identity fit would be moderated by holisticness: We expected an interaction between holisticness and self-definingness, whereby only highly holistic groups (Christianity, Healthy-living, Environment) would show positive relations between self-definingness and inter-identity fit (i.e., less holistic groups (Gender, Nationality) would not).

Bivariate correlations (see Table 1) provide support for this expectation: Self-definingness was generally more strongly related to inter-identity fit for the three groups which were expected to be high in holisticness and more weakly related in those low in holisticness. For example, Christianity shows a moderate to strong relation between its self-definingness (Table 1, row 10) and inter-identity fit (column 12), while gender shows no discernible relation between its self-definingness (row 2) and inter-identity fit (column 4). For a discussion of bivariate results concerning the relation between identification and self-definingness see the methodological section in S1 Appendix.

**Table 1:** Bivariate correlations of identity variables for all five groups.

		2	3	4	5	6	7	8	9	10	11	12	13	14	15	16	17	18	19	20
Gender																				
1	Holisticness	.75**	.29**	.19*	.51**	.45**	.23*	.18	.11	.18	.17	.23*	.18	.38**	.20*	.22*	.09	.25**	.19*	.24*
2	Self-definingness		.50**	.07	.48**	.53**	.42**	.12	.20*	.30**	.26**	.12	.20*	.32**	.17	.12	.11	.25**	.24*	.17
3	Identification			.05	.14	.31**	.33**	.11	.11	.27**	.27**	.01	−.05	.17	.22*	.06	−.03	.24*	.28**	.24*
4	Identity fit				.09	.09	.05	.49**	.06	.19*	.23*	.45**	.17	.23*	.20*	.60**	.07	.23*	.24*	.55**
Nationality																				
5	Holisticness					.73**	.50**	.22*	.09	.18	.10	.11	.06	.17	−.10	.23*	.10	.02	−.06	.26**
6	Self-definingness						.65**	.36**	.23*	.47**	.40**	.17	.03	.21*	.03	.26**	.01	.16	.13	.35**
7	Identification							.29*	.32**	.50**	.48**	.24**	.01	.12	.07	.27**	−.02	.03	−.01	.28**
8	Identity fit								.33**	.40**	.43**	.49**	.14	.18	.13	.65**	−.10	.11	.13	.57**
Christianity																				
9	Holisticness									.62**	.62**	.40**	.10	.06	−.06	.03	.12	-.02	−.04	−.03
10	Self-definingness										.95**	.51**	−.01	.17	.10	.21*	−.12	.07	.08	.19*
11	Identification											.55**	.01	.15	.12	.23*	−.13	.08	.11	.18
12	Identity fit												.16	.04	.03	.37**	−.03	−.08	−.04	.25**
Healthy-living																				
13	Holisticness													.55**	.43**	.08	.46**	.28**	.29**	−.01
14	Self-definingness														.74**	.19*	.25**	.50**	.50**	.28**
15	Identification															.19*	.15	.45**	.57**	.23*
16	Identity fit																−.06	.14	.20*	.74**
Environmental																				
17	Holisticness																	.47**	.33**	-.01
18	Self-definingness																		.80**	.31**
19	Identification																			.37**
20	Identity fit																			

* *p* < .05,

** *p* < .01

In order to test our hypothesis more directly, we conducted multi-level regression, regressing a dummy variable of holisticness (0 = low holisticness (Gender, Nationality), 1 = high holisticness (Christianity, Healthy-living, Environment); step 1), mean centred self-definingness (step 2) and the interaction between holisticness and self-definingness (step 3) on our dependent variable—inter-identity fit—while controlling for gender and age (both of which were non-significant). A multi-level (random-intercept) regression was conducted in MLwiN (version 2.23) to control for the repeated measures design (i.e., participants answered the questions for all five groups). The intraclass correlation indicated that 50.37% of variance occurred at the participant-level, reinforcing the importance of this multi-level control.

Regressions confirm the hypothesized interaction: Holisticness (B = 0.09, SE = 0.08, *t* (554) = 1.17, *p* = .12) was only related to overall group fit through its interaction with self-definingness (B = 0.10, SE = 0.50, *t* (554) = 1.92, *p* < .03). There was also a main effect of self-deiningness (B = 0.10, SE = 0.50, *t* (108) = 2.18, *p* < .02), indicating that—somewhat unexpectedly -self-definingness does still have some positive association with inter-identity fit for groups low in holisticness (which again seems primarily attributable to the more holistic nature of Nationality than initially expected). Notably however, this association is significantly higher for groups high in holisticness. The overall fit of the model was good, in comparison to a model with only controls entered (*χ* ^2^_difference_ (3) = 42.88, *p* < .001). Thus, as expected, the extent holistic philosophies are applied to life (i.e., self-definingness) was central to the ability of holistic groups to promote inter-identity fit.

### Discussion

Results confirmed our expectation that there is substantial variation between groups in the extent to which they (a) holistically prescribe a philosophy for life, and (b) are actually used across all life contexts in this self-defining way. Moreover, the fit between multiple social identities was influenced by a combined effect of self-definingness and holisticness: When an identity was both holistic and strongly self-defining, it fitted more strongly with multiple (otherwise unrelated) social identities. In other cases, when either the identity was less holistic and/or it was more weakly self-defining, different aspects of the self fitted together more weakly. Interestingly, holisticness appears to be a quality of group philosophies which can be either self-selected (e.g., Christianity) or ascribed (e.g., Nationality). Moreover, we provide important preliminary evidence that Environmental and Healthy-living groups (in addition to religion) provide philosophies for life and are also associated with stronger inter-identity fit (although less strongly than religion). This suggests it is the group’s ideologies and the way they are personally applied which is key to promoting inter-identity fit.

Findings strongly and consistently identified two groups that best characterize more and less holistic identities: Christianity and Gender respectively. Results indicate that although gender carries great personal significance and meaning, in modern society (at least in this culturally Western sample) its social identity content does not generally prescribe its members with a particular philosophy to follow that truly guides their life (e.g., determines personal goals), in the same way that Christianity does (e.g., promoting inter-identity fit). Importantly for this research, gender and Christianity are orthogonal identities (i.e., being a woman says nothing about one’s religion), so their fit can be attributed to the individual’s representation of the identity. Moreover, because gender is naturally occurring (i.e., not personally chosen) variation in fit between religious and gender identities should be induced by the use of holistic group philosophies, not pre-existing similarities or differences between identity content. Consequently, gender and Christianity are expected to be a good starting point for our analysis, and will be utilized in the proceeding studies. Notably, these studies focus on the female gender identity only. There were methodological and theoretical reasons for this. Methodologically, if more than one gender were used, the differing contents of female and male identities means that they could not be aggregated nor even directly compared in studies 2 and 3. Theoretically, it has often been found that women tend to consider gender a more central aspect of their self-concept (e.g., [50]) and thus we thought it likely that cleaner and clearer effects may be achieved in female populations. Thus, holding these group factors constant allowed us to focus on the application of different measurement methods in studies 1–3 in order to demonstrate convergent evidence for our hypothesis.

## Study 1

Study 1 consisted of a questionnaire with self-report measures to test the relationship between self-definingness and participants personal perceptions of fit between gender and religious identities. We predicted that the stronger the self-definingness of religion (a group high in holisticness) the stronger its fit with gender identity. Conversely, we do not expect the self-definingness of gender (a group lower in holisticness) to have consequences for its fit with religion.

### Method

#### Sample

One-hundred-and-thirty-four female students from Groningen University (age: 17–29; M = 19.48, SD = 2.18) participated in exchange for course credit. Forty-eight women were religious and 68 were not (of this 11 continued to identify with a branch of Christianity; for theoretical and statistical motivations of this choice, see S5 Appendix), and 16 were uncertain (of whom 15 identified with a form of Christianity). The remaining two participants did not specify their religious status.

#### Design

Independent variables were self-definingness of gender and religion, as assessed through a self-definingness scale. Dependent variables were measures of inter-identity fit.

#### Procedure

The questionnaire was computer-administered in isolated cubicles, with materials programmed in Authorware 7.0. All participants were presented with a range of statements with which they were asked to indicate their agreement on 7-point Likert-type scales (1 = strongly disagree, 7 = strongly agree). The full version of the inter-identity fit and self-definingness scales developed for the Pilot were used in Study 1, due to their central importance. Thus, a number of additional items were included for each scale.

Seven items measured *self-definingness*. Reliabilities were very high for both religion (*α* = .97) and for gender (*α* = .83). A factor analysis confirmed that self-definingness is a single factor, and provides firm evidence of convergent and divergent validity (see methodological section of S1 Appendix).

*Inter-identity fit* was measured using a five-item scale designed to assess the inter-identity fit of Christian with female identity (*α* = .66) and vice versa (*α* = .76). This scale focuses on the consciously perceived overlap between two distinct social identities in terms of experienced fit between normatively accepted values, principles and behaviours.

Finally, participants reported their age, gender, religion and whether they were religious (trichotomous response; yes, no, or unsure), before being thanked and debriefed.

### Results

We expected that only self-definingness of religion (as more holistic) would be associated with stronger fit among social identities. In order to test this hypothesis separately, simple regressions were carried out with the outcome variables of inter-identity fit predicted by, (1) self-definingness of religious identity, and (2) self-definingness of gender identity. Age was entered as a control.

First, regressions using self-defining religiosity as a predictor revealed self-definingness of religion was strongly, positively associated with inter-identity fit: The more self-defining one’s religious identity, the higher the perceived fit of religion in gender (B = 0.21, SE = 0.04, β = .39, 95% CI: 0.23, 0.55; *t* (129) = 4.91, *p* < .0001) and gender in religion (B = 0.19, SE = 0.04, β = .32, 95% CI: 0.18, 0.51; *t* (129) = 4.23, *p* < .001).

Second, regressions using self-defining gender as a predictor showed that it was not, across the board, strongly associated with inter-identity fit: Indeed, it was not as strongly related to religion’s fit in gender (B = 0.16, SE = 0.09, β = .16, 95% CI: -0.01, 0.33; *t* (129) = 1.86, *p* > .06) or the fit of gender into one’s religious identity (B = 0.21, SE = 0.09, β = .20, 95% CI: 0.03, 0.37; *t* (129) = 2.36, *p* < .03), although effect sizes were notable.

Reviewing findings on the basis of effect sizes (95% confidence intervals calculated around standardized betas using the “Methods for the Behavioural, Educational and Social Sciences” Package in R [51]), we can see that self-definingness of gender has relatively little influence on inter-identity fit, but self-definingness of religiosity had relatively strong influence. Specifically, self-defining religiosity is not only associated with religious aspects of an individual’s identity, but also has a strong positive association with inter-identity fit, bidirectionally informing the fit of religion in gender.

### Discussion

We found that the self-definingness of religion and gender influenced inter-identity fit largely as expected. The self-definingness of religious identity was strongly associated with its fit with the social identity of gender: Crucially, the more self-defining religious identity was, the greater the perceived fit between it and female identity. Although, the self-definingness of gender identity was more strongly associated with perceptions of inter-identity fit than initially predicted, in line with the expectations, the effects of religion were much stronger. Thus, self-definingness of religious identity was reliably associated with inter-identity fit.

## Study 2

In this study, we used a more implicit measurement of inter-identity fit than in Study 1. We adapted a standard experimental paradigm designed to investigate cognitive inter-identity fit (first developed by Aron et al. [44], and extended by Smith & Henry [45]; for notes about our adaptation, see S6 Appendix). The procedure first identifies, for each individual participant, what traits are associated with the self, and with gender and religious identities. Then, response times (RTs) are recorded for participants’ judgments of the extent to which traits describe the self (i.e., a split-second yes/no decision). Finally, the RTs on traits that match self, gender and religion are compared with those that mismatch. Prior research [44, 45, 52] indicates that when these identities fit together—with overlapping cognitive representations—a match speeds up RTs while a mismatch slows RTs. Thus, this paradigm allows us to assess the consequences of self-definingness for inter-identity fit more implicitly, using RTs as an indicator of positive and overlapping cognitive representations of multiple social identities. We predicted that individuals with a stronger self-definingness of religious identity would be associated with faster responses to traits that match both social identities but more slowly to mismatching traits. In contrast, self-definingness of gender should be unrelated with RTs to traits (mis)matching multiple social identities.

### Method

#### Participants

Two-hundred-and-one females (age: 18–88; M = 32.71, SD = 12.55) were recruited via Mechanical Turk, participating in exchange for ¢50. In order to achieve a representative sample the religious aspect of the study was not mentioned in the title or study description which people read before electing to participate. Sixteen individuals were excluded from the analysis due to their identification with non-Christian religious beliefs (e.g., pagan and Islamic beliefs). A further 4 participants were excluded due to unsuccessful completion of the study, leaving a final sample of 181 participants: 83 self-classified as Christian, and 95 as non-religious (28 of which continued to identify with a denomination of Christianity). The remaining 3 participants did not specify.

#### Materials

The study was completed individually on a computer. It was conducted over the internet and composed on Adobe Flash (version 3). Reliabilities of RTs measured using Adobe Flash were piloted and found to be adequate. Indeed, prior research has employed similar online methodologies [53] and found them to be reliable [54].

The list of 90 traits (required for the Aron et al. [44] and Smith & Henry [45] methodology) was composed of 70 identity-concepts and a further 20 control items. To maximize the possibility of the measurement of both matches and mismatches of the self with both women and Christian identities, of the 70 identity-concepts, 25 were uniquely associated with women (e.g., nurturing, multi-tasker), 25 were uniquely associated with Christians (e.g., believers, devoted) and 20 were shared between female and Christian identities (e.g., loving, helpful). The trait lists were drawn from a list of traits associated with both identities by a separate but comparable sample (see Study 3), selecting those items for which there was a reasonable degree of consensus, without too much overlap. Control items (e.g., sporty, adventurous) were included to minimize suspicion and to encourage matches across identity associates.

#### Procedure

The study was entitled ‘group descriptions’ and was introduced as a questionnaire about the words associated with the self and particular groups. Participants clicked a link to access the study. First they completed the trait descriptiveness task (cf. [45]), indicating to what extent 90 traits were descriptive of themselves and of the general characteristics of their religious and gender group on 7-point scales (1 = extremely unlike, 7 = extremely like). The order of the three sets was randomized, as were traits.

Participants then completed the response latency task. After a practice session, in which five neutral example traits (e.g., healthy) were presented, the 90 target traits were presented randomly. They had to make dichotomous (yes/no) decisions whether traits described the self (S-key) or not (L-key). Participants were instructed to keep their forefingers on the keys throughout. Stimuli were prefaced by a fixation point (XXXX), presented centre-screen for 1,000 ms. Subsequently, a trait was presented until the subject pressed one of the keys. Participants were then given a rest period of 1,000 ms before the next sequence started.

Next, participants completed a questionnaire including the self-definingness measures (see Study 2). Furthermore, in order to counteract any potential acquiescence bias present in the self-definingness scale, three additional negative items were reversed scored and included in the Christian (*α* = .72) and gender scale (*α* = .91; e.g., My gender doesn’t say a lot about the real me). Finally, participants were thanked and debriefed.

#### Data cleaning

RT data was cleaned according to procedures presented by Wentura and Degner [55]. RTs below 300 ms were considered to represent anticipations, not actual conscious responses and were consequently excluded. RTs above 10,000 ms were also excluded as they were considered to signal insufficient task-oriented attention. Furthermore, all responses 3 standard deviations above or below the mean were removed. In this way, 4.38% of RTs were excluded, comfortably within the frequent outlier occurrence rate (0.29%– 5.80%) reported by Wentura and Degner [55] or the 10% rate suggested by Ratcliff [56]. Finally, RTs were subjected to a natural logarithm transformation to correct their classically skewed distribution.

The main task revealed four Match-types—each match type consists of two equivalent response patterns. For example, there are traits that are self-descriptive, and which are also associated with Christianity and gender (‘Self-Identities Match’). This response pattern is (theoretically) equivalent to its inverse where traits are not self-descriptive, and are not associated with gender or Christianity. From a theoretical perspective we expect that highly self-defining Christians respond quickly to both patterns. Similar match types can also be constructed for ‘Self-Gender Identity Match’ (traits are (non) descriptive of the self and gender traits but not Christians), ‘Self-Religious Identity Match’ (traits are (non) descriptive of the self and Christians but not gender), and ‘Self-Identities Mismatch’ (traits which do (not) describe the self are (not) non descriptive of gender and Christianity). We statistically tested for differences between each pair of (inverted) response patterns, but none were found (all *p*’s > .05). This confirms that collapsing into four Match-Types did not distort the pattern of results.

### Results

#### Self-trait associations: Response latencies

The data was analyzed using multi-level analysis conducted in MLwiN (version 2.18). This analysis takes into account that RTs are nested within the individual.

A random intercept model (allowing individuals to differ randomly in their average RT) was built. In the first step, the control variable of age was entered. In the second step, three categorical independent variables–‘Self’, ‘Christian’ and ‘Women’- were entered. They indicated whether specific traits were descriptive of Christians and women, (dichotomized so that responses from 1 to 3 were coded as ‘no’, those ranging from 5 to 7 were coded ‘yes’, with 4 coded as missing: see [45], [52]) and of the self (using the dichotomous yes/no response to the response latency task). Each independent variable was dummy coded 0 (no; reference category) and 1 (yes). In the third step, the Match-Types were entered (with Self-Identities Mismatch as the reference group). Step four included individual-level continuous variables of self-defining religion and gender (centered). Finally, step five comprised the interaction between self-defining religion (centered) and Match-Types, and similarly step six included the interaction between self-defining gender (centered) and Match-Types.

An initial inspection of independence and normality of residuals suggested that our final model was an acceptable fit to the data. Intraclass correlation indicated that a substantial 30% of the variance was accounted for by between-individual differences in RTs [57]. This confirms the need for a multi-level analysis. The final model is presented in Table 2.

**Table 2:** Summary of Multi-level regression model predicating reaction times for trait classifications.

	Coefficient	S.E.	Increase in Model fit
Intercept	6.95	0.021	
Step 1 (control variables)			*Χ* ^*2*^ (1) = 101.792**
Age	0.001	0.001	
Step 2			*Χ* ^*2*^ (3) = 40932.21**
Self	−0.127***	0.011	
Christian	−0.029*	0.010	
Women	−0.014	0.011	
Step 3			*Χ* ^*2*^ (3) = 32.82**
Self-Gender Identity Match	−0.060	0.016	
Self-Religious Identity Match	−0.059*	0.016	
Self-Identities Match	−0.074***	0.013	
Step 4			*Χ* ^*2*^ (2) = 2.34
Self-defining religion	0.015	0.009	
Self-defining gender	−0.011	0.013	
Step 5			*Χ* ^*2*^ (3) = 41.66**
Self-defining religion x Self-Gender Identity Match	−0.009	0.007	
Self-defining religion x Self-Religious Identity Match	−0.024*	0.008	
Self-defining religion x Self-Identities Match	−0.029***	0.005	
Step 6			*Χ* ^*2*^ (3) = 3.10
Self-defining gender x Self-Gender Identity Match	−0.013^	0.009	
Self-defining gender x Self-Religious Identity Match	0.003	0.011	
Self-defining gender x Self-Identities Match	−0.002	0.007	

^^^ *p* < .08,

* *p* < .05,

** *p* < .001,

*** *p* < .0001

Our focal prediction was that there would be a significant interaction between Match-Type and self-defining religiosity. Naturally, we expect that traits associated with self and Christianity are responded to more quickly among individuals with stronger self-defining religiosity. However, the critical test of the hypothesis is if stronger self-definingness of religiosity predicts faster responses to traits with a full-match between the self and both one’s ingroups (i.e., overlap between all three identities). This interaction would reflect the stronger cognitive overlap of multiple social identities within the self. In contrast, self-definingness of gender was expected to be unrelated to all RTs.

**Fig 2:**
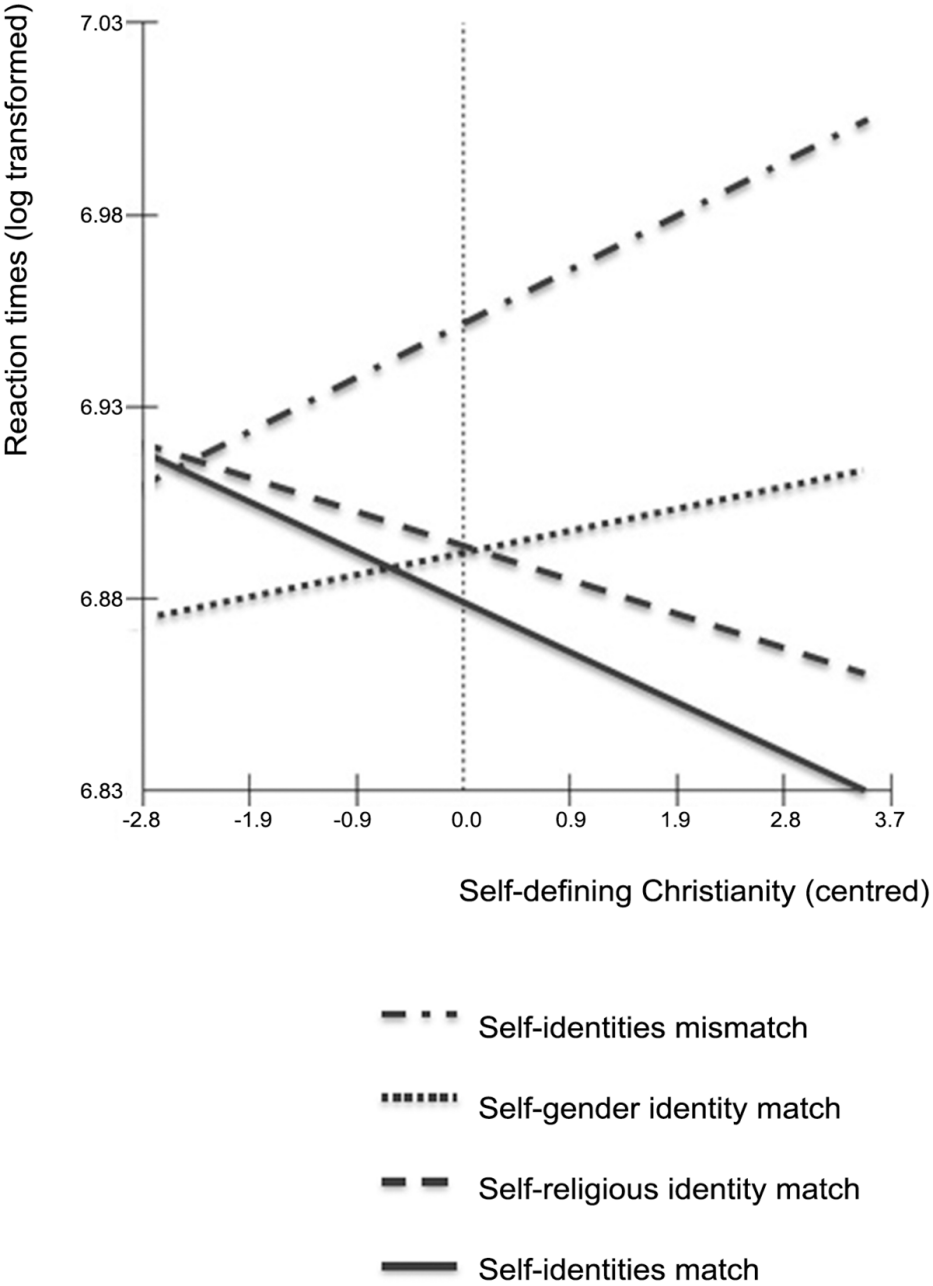
Log transformed reaction times representing the interaction between match-type and self-defining Christianity (centred).

Results show a main effect of Match-Type. Crucially, this was qualified by the predicted interaction between Match-Type and self-defining religion (see Fig 2). The increase in model fit is highly significant (*χ* ^2^_difference_ = 41.66, p < .001), which indicates significant differences between slopes. Moreover, the direction of the interaction confirms predictions. In line with expectations, the Self-Identities Match slope is steepest and significantly negative (B = -0.029, SE = 0.005, *t* (8781) = 5.89, p < .0001). So, the more religion is self-defining for an individual, the quicker they responded to traits that overlap across all three identities. Notably, the Self—Religious Identity Match slope was also significantly quicker (B = -0.024, SE = 0.008, *t* (8781) = 3.00, p < .05). However, no significant effect of Self—Gender Identity Match was found.

The significant interaction was followed up with tests of differences between slope estimates (see Table 3). Results indicate that overlap between self and Christian traits is strongly associated with the facilitation of fast RTs. Although adding an additional overlap with women to this is not associated with significantly faster RTs (i.e., Self—Religious Identity Match does not differ significantly from a Self—Identities Match), important substantive differences exist between these two Match-Types. Self—Identities Match differs from both Self—Identities Mismatch and Self—Gender Identity Match, while Self—Religious Identity Match only differs significantly from the former. So, Self—Identities Match differs strongly and consistently from other Match-Types, while Self—Religious Identity Match occupies an intermediary position. Thus, although overlap between the self and one’s religious identity appears to be (unsurprisingly) important for strongly self-defining Christians, the additional overlap with the ancillary identity of gender may be substantively important for the self.

**Table 3:** Contrasts between beta estimates of the interaction between different Match-Types and self-defining religion.

Match-Type		1	2	3	4
1	Self-Identities Mismatch	-	1.71	10.03*	32.67**
2	Self-Gender Identity Match		-	3.69	12.35**
3	Self-Religious Identity Match			-	0.51
4	Self-Identities Match				-

* *p* < .05,

** *p* < .001. Contrast are tested using a Chi-Square distribution, with one degree of freedom

As expected, the interaction between Match-Type and self-defining gender was not significant. Notably, a marginally significant effect of self-defining gender with Self—Gender Identity Match emerged (B = -0.013, SE = 0.009, *t* (8781) = 1.44, *p* = .07), indicating that stronger self-definingness of gender is associated with faster responses to traits overlapping with the self and the female identity (in comparison to a Self-Identities Mismatch). No other significant results were found.

In sum, inspection of between-slope differences confirms that self-definingness of religion is associated with—not only for the inclusion of one’s religious identity into the self—but also the cognitive overlap in the representation of the self and religious identity with the ancillary identity of gender. In contrast, although self-definingness of gender may be associated with the fit between one’s gender identity and the self, it shows no substantive association with its fit with the ancillary religious identity.

#### Self-trait associations: Content analysis

We then assessed whether self-definingness influences not only the speed of responses but also identity content (i.e., the trait-descriptiveness judgments made). We hypothesized that stronger self-definingness predicts a higher proportion of trait classifications as overlapping between all three identities (i.e., matching the self, women and Christian), and fewer errors. Errors were coded as responses during the response latency test that disagreed with earlier questionnaire responses regarding the self-descriptiveness of traits.

First, simple regressions were conducted using self-defining religiosity as a predictor. Age was entered as a control variable. The proportion of (a) traits classified and (b) errors made within each of the four Match-Types served as dependent variables in separate regressions. Due to the number of post-hoc test conducted Bonferroni correction was applied, testing at the more stringent *p* < .003 (i.e., *p* = .05/16 tests).

Self-definingness of religion was inversely related to Self-Gender Identity Match (B = -0.04, SE = 0.01, β = -.53; *t* (171) = 8.05, *p* < .0001). Unsurprisingly, the more strongly self-defining classified fewer traits as overlapping with their personal and gender identity, but not within their religious identity. In direct support of our focal prediction, self-definingness was positively related to the proportion of Self—Identities Match responses (B = 0.05, SE = 0.01, β = .43; *t* (171) = 6.34, *p* < .001). The more self-defining one’s religion, the more traits were listed as overlapping with all three aspects of one’s identity.

The error analysis showed only a significant effect for Self—Identities Mismatch (B = − 0.05, SE = 0.01, β = −.31; *t* (152) = 3.95, *p* < .0002), whereby the more self-defining one’s religious identity, the more incorrect answers were given for traits that were non-overlapping between the self and both ingroups. Thus, self-defining religion had clear and consistent consequences for inter-identity fit.

These analyses were repeated using self-defining gender as the predictor variable. However, no significant effects were found at our Bonferroni corrected level (although effects sizes of models predicting proportion of Self—Identities Match responses and Self-Gender Identity Match were notable they were substantively smaller than those predicted by self-defining religion; B = 0.04, SE = 0.01, β = .21; *t* (171) = 2.94, *p* > .003; B = -0.02, SE = 0.01, β = -.19, *t* (171) = 2.57, *p* >.01 respectively). We therefore conclude that the association between self-defining religion and ancillary social identities within the self-concept is not replicated as a function of the self-definingness of gender. Thus, in contrast to self-defining religion, self-defining gender is largely irrelevant to the fit between multiple social identities.

### Discussion

In Study 2, we replicated support for our hypothesis using more implicit measures. An RT analysis revealed that religiosity had consequences for cognitive representations of inter-identity fit and judgment processes, influencing both the speed and type of self-descriptiveness judgments made. In line with predictions, cognitive overlap between the self and one’s religious and gender identities was stronger at higher levels of self-definingness; evidenced by the stronger facilitation of responses to traits overlapping between these three identities, and inhibition of non-overlapping traits. Building upon previous findings [45, 52], Study 2 therefore shows that overlap can exist in the representation of the self and multiple social identities. Moreover, self-definingness of the more holistic religious identity moderates this cognitive fit.

In contrast, self-definingness of the less holistic gender only had consequences for that specific social identity. Although subjectively people report levels of self-definingness for gender and for religion that are (on average) equally high (see Pilot), gender’s self-definingness does not appear to regulate the fit among different aspects of self in the same way that religion does. Indeed, we have found only limited evidence of an association between inter-identity fit and self-definingness of gender using both explicit self-reports (Study 1) and more implicit (RT) measures tapping into cognitive representations (Study 2).

## Study 3

This study aimed to further examine identity contents by focusing on *self-generated* identity content, rather than identity content pre-selected by researchers. We therefore modelled participants’ idiosyncratic perceptions of their religious and gender identities in order to build a ‘cognitive map’ of inter-identity fit. We could then use a novel network analysis to test the extent to which self-definingness of religion and gender is associated with strength of inter-identity fit between religious and gender identities.

To this end, we developed a new network-based measure of identity content that relies on the Associative Recall Task (ART). In this task, subjects are instructed to freely recall words (see also [58]) that they personally associate with certain social identities (in the present research: gender and religion; see also [59]). Semantic network analysis can represent and systematically test this identity content by generating a ‘cognitive map’ which structures this content on the basis of relations (links) between identity content (nodes) recorded within participants’ lists [60]. Such an analysis allows the examination of inter-identity fit, not only in terms of micro-level overlap between individual traits, but also macro-level inter-identity overlap between multiple social identities. Together, ART and the semantic network analysis facilitate an organic investigation of an individual’s representation of the fit between the identity content of multiple identities. We predicted that the more self-defining one’s religious identity the more overlapping gender and religious identity content will be. In contrast, we expect self-definingness of gender to be unrelated to overlap between religious and gender identities.

### Method

#### Participants

This sample consisted of 50 females (age 18–64; M = 34.37, SD = 13.68) from the United States, recruited from Mechanical Turk in exchange for $1. As in Study 2, the religious aspect of the study was not mentioned in the title or study description which people read before electing to participate. Five participants were removed from the sample because they were of a non-Christian faith. A further two participants were removed due to invariant answer patterns, leaving a final sample of 43 women. In the final sample, the majority self-identified to some extent as Christian (N = 34). Nine participants indicated being non-religious.

#### Procedure

The study was completed individually on a computer over the Internet. It was entitled ‘group traits questionnaire’ and was introduced as an investigation into the words associated with particular groups. It was composed of two phases; the ART and a questionnaire. The study itself was composed using eXamine (version 2.0), a web survey tool for internet research.

#### Phase 1: Associative Recall Task

Participants read instructions which asked them to list characteristics, traits, attributes, and qualities they considered to be important or centrally defining of two different social targets or that distinguished these targets from other people. Participants then practiced with an example; an athlete. They were given three examples words associated with athletes (sporty, athletic, healthy) followed by a maximum of five spaces in which to write their own associations. This practice section aimed to encourage both appropriate and fluid associations by familiarizing participants with the task. Importantly, the target of ‘athlete’ was chosen because it was largely independent of the subsequent targets (women and Christian). Following the completion of the practice items, participants were given twenty spaces in which to write words they associated with women and, on a separate screen, Christians. The order was counterbalanced.

#### Phase 2: Questionnaire

Several questions assessed inter-identity fit between the trait-level content of female and Christian identities by exploring the degree of personally experienced association among contents of the ART lists. Participants indicated agreement with statements on Likert-type 7-point scales (1 = strongly disagree, 7 = strongly agree).

Five adapted items based on the inter-identity fit scale sought to assess *inter-identity content fit*, in how strongly associated the women and Christian lists were overall (e.g., the traits in list 1 are consistent with the traits in list 2; *α* = .68). Additionally, the normalized number of *traits repeated* across both (female and Christian) lists was recorded (normalized to correct for the different number of traits written on the ART lists) as a measure of positive association between gender and religious identities in terms of actual the overlap in the content or meaning they are perceived to prescribe. (Notably, this measure was compiled after the content cleaning, see below).

Finally participants responded to the seven-item *self-definingness* scale for the Christian (*α* = .98) and female (*α* = .92) identities.

#### ART responses

ART responses were subject to a cleaning and generalization procedure. We corrected for spelling errors and merged concepts that were identical but recorded differently (e.g., ‘compassionate’ and ‘have compassion’) or were semantically synonymous (e.g., ‘petite’ and ‘small’, for a full list see Table A in S7 Appendix).

In order to facilitate the comparison of strongly and weakly self-defining religion and gender, four semantic networks were generated. First, two networks were created based on the self-definingness of religion. The sample was split at the median (see “Median Split” in S7 Appendix) (Mdn = 4.29) to form one strongly (n = 22; concepts listed = 496; shared concepts = 59%; unique concepts = 41%) and one weakly (n = 21, concepts listed = 448; shared concepts = 47%; unique concepts = 50%) self-defining network. Next, two networks were created based on the self-definingness of gender, with one strongly (n = 22; concepts listed = 487; shared concepts = 53%; unique concepts = 47%) and one weakly (n = 21; concepts listed = 457; shared concepts = 52%; unique concepts = 48%) self-defining network, which were also formed via a median split (Mdn = 4.86). Networks were generated using Automap (version 3.0.7), a text analysis software package. Each network consisted of a matrix demarcating the dichotomous presence or absence of a link between concepts. Links were coded between concepts on the same list on the basis of a windowing method (see “Window Method” in S7 Appendix). This window moves through each participant’s list coding a link between every concept (either a single word (e.g., strong) or a set of words (e.g., family orientated)) with three adjacent concepts.

### Results

**Fig 3:**
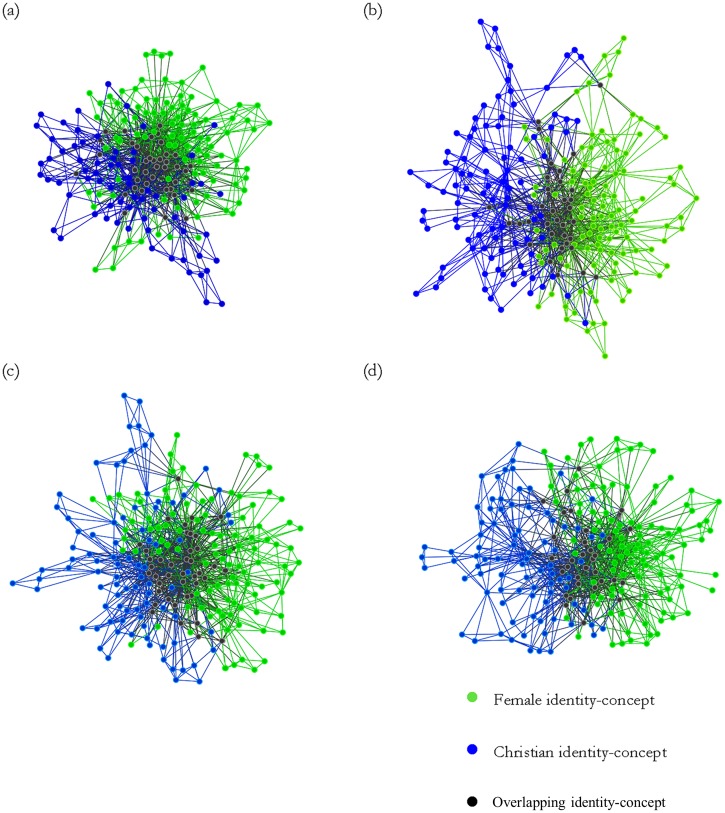
Aggregated networks of traits associated with female and Christian identities for (a) strongly self-defining Christians; (b) weakly self-defining Christians; and (c) strongly self-defining women; and (d) weakly self-defining women.

This analysis investigated the association between the self-definingness of gender and Christianity and the fit between the content of these two social identities. The content and structure of (strongly and weakly self-defining) Christian and gender identities were compared and contrasted by conducting a semantic network analysis on the ART lists. All networks and relevant descriptives are presented in Fig 3 and Table 4, respectively.

**Table 4:** Network descriptives of both the strongly and weakly self-defining networks for (left) religion and gender (right).

	Religion Networks			Gender Networks		
Network characteristic	Strongly self-defining (S)	Weakly self-defining (W)	Difference (S-W)	Strongly self-defining (S)	Weakly self-defining (W)	Difference (S-W)
Density	.065	.044	.021	.050	.052	.002
Diameter	5.00	7.00	2.00	6.00	6.00	0.00
Proportion of Nodes						
Women	.471	.453	.024	.446	.472	-.026
Christian	.338	.403	-.069	.377	.376	.001
Overlapping	.191	.144	.045	.177	.151	.026
Proportion of Links						
Women & overlapping	.178	.160	.018	.171	.165	.006
Christian & overlapping	.139	.106	.033	.128	.122	.006
Women & women	.262	.308	−.046	.252	.305	−.053
Christian & Christian	.177	.266	−.089	.217	.235	−.019
Overlapping & overlapping	.244	.160	.084	.232	.173	.060

#### Network shape and content

As can be seen in panel (a) of Fig 3, the network for strongly self-defining Christians was highly interconnected (see “Figure notes” in S7 Appendix). The density suggests better connectedness than in any other network (see Table 4); a finding which is reinforced by the small network diameter (presenting the shortest possible number of links that must be crossed when moving between the two most distant nodes; see Table 4, left half).

Looking beyond network shape to content, female and Christian concepts are also highly integrated in the networks of strongly self-defining Christians. Not only do a substantial proportion of links bind uniquely female and Christian concepts to overlapping concepts (i.e., concepts which occurred on both Christian and female lists), but Table 5 indicates that the central nodes within this network are overlapping identity-concepts. Measures of degree, closeness, and betweenness converge to reveal ‘strong’, ‘caring’, ‘hard-working’ and ‘loving’ as key hubs. These nodes are adjacent to over half the concepts in the network, have the closest average distance to other concepts in the network, and help bridging concepts that would otherwise be unconnected. In this way, even uniquely female and Christian concepts are bought into close proximity, suggesting strong positive association. Thus, the content of female and religious identities of strongly self-defining Christians appears to be very closely intertwined.

**Table 5:** Strongly self-defining (left) and weakly self-defining (right) Religion network descriptives for important identity concepts in each network.

Strongly self-defining Religion						Weakly self-defining Religion					
Word	List	Frequency	Betweenness	Closeness	Degree	Word	List	Frequency	Betweenness	Closeness	Degree
Strong	Overlaps	20	.13	.55	.60	Strong	Female	15	.33	.50	.48
Caring	Overlaps	19	.13	.57	.60	Smart	Overlaps	11	.21	.45	.40
Hardworking	Overlaps	12	.11	.54	.51	Kind	Overlaps	12	.20	.48	.42
Loving	Overlaps	19	.11	.58	.61	Believers	Christian	5	.19	.42	.25
Helpful	Overlaps	10	.09	.51	.39	Religious	Christian	9	.19	.45	.33
Compassionate	Overlaps	13	.08	.53	.44	Nurturing	Female	5	.16	.42	.25
Intelligent	Female	8	.08	.51	.37	Beautiful	Female	9	.13	.45	.36
Understanding	Overlaps	7	.04	.48	.30	Dependent	Overlaps	2	.10	.38	.12
Faithful	Christian	7	.04	.50	.32	Good	Overlaps	5	.10	.44	.23
Nurturing	Female	5	.04	.49	.28	Wives	Female	3	.09	.38	.13

In contrast, the network of participants who are weakly self-defining Christians is more diffuse, as indicated by the deflated density and inflated diameter. Moreover, the weakly self-defining network shows a fairly clean segregation between uniquely female and Christian traits; few of which are connected via overlapping concepts. Indeed, some of the more central traits hubs within this network tend to be uniquely Christian and female, (e.g., ‘believers’, ‘religious’ and ‘strong’, ‘nurturing’, respectively; see Table 5). This indicates that these nodes are important bridges between other uniquely religious or female concepts and the rest of the network, which correspondingly emphasizes the segregation between these groups of identity concepts. Notably, the higher betweenness scores of the weakly self-defining network (in comparison to the strongly self-defining) suggests that overall connectedness of the network is lower, in that the network would be more sensitive to the breakdown in connectedness of a few key concepts. So, although some joint concepts are shared between women and Christians, the links within the lists are much stronger and more robust than those overlapping between them.

Interestingly, the node ‘strong’ is a uniquely female attribute amongst weakly self-defined Christians, but an overlapping concept in the strongly self-defined Christians (see “Overlap Coding” in S7 Appendix). It seems poignant that strongly self-defining individuals bridge these two identities using a concept that is most central to the *female* identity. This is an interesting indication that the high degree of interconnection among strongly self-defined Christians is not merely a matter of Christian values dictating gender identity: there may also be a recursive influence of gender characteristics informing Christian identity in a bi-directional interaction.

Table 4 (right half) shows that the networks of strong and weakly self-defining gender participants are highly comparable to each other. Similarly, Table 6 indicates that the key concepts and their descriptives are not substantively different for both gender networks. The characteristics of these two networks fall somewhere in between the religion networks (which is unsurprising as they are based on the same data). The lack of difference between the two networks shows that self-definingness of gender has little consequence for the fit between gender and religious identity-concepts.

**Table 6:** Strongly self-defining (left) and weakly self-defining (right) gender network descriptives for important identity concepts in each network.

Strongly self-defining Gender						Weakly self-defining Gender					
Word	List	Frequency	Betweenness	Closeness	Degree	Word	List	Frequency	Betweenness	Closeness	Degree
Strong	Overlaps	18	.15	.53	.54	Strong	Overlaps	17	.14	.51	.43
Smart	Overlaps	13	.11	.50	.40	Hardworking	Overlaps	13	.13	.51	.48
Beautiful	Christian	12	.10	.51	.39	Loving	Overlaps	14	.11	.53	.45
Caring	Overlaps	14	.09	.53	.44	Helpful	Overlaps	9	.09	.48	.36
Kind	Overlaps	13	.07	.50	.40	Faithful	Christian	7	.09	.50	.32
Intelligent	Female	7	.07	.46	.25	Religious	Christian	6	.08	.46	.26
Compassionate	Overlaps	7	.06	.43	.24	Nurturing	Female	6	.07	.45	.28
Loving	Overlaps	11	.06	.49	.35	Caring	Overlaps	9	.07	.48	.32
Religious	Christian	8	.05	.45	.22	Understanding	Overlaps	7	.06	.46	.29
Believers	Christian	6	.05	.44	.23	Compassionate	Overlaps	10	.05	.49	.33

#### Statistical network comparisons

Exponential Random Graph (ERG; [61]) Models enable us to go beyond descriptions of network and content and to conduct statistical tests of between-network differences. ERG models investigate the micro foundations of macro network tendencies (i.e., what node-level tendencies produce the observed network characteristics). They recreate a population of networks similar to the observed network through a stochastic process in which the formation of a link has both a stochastic component as well as (parameterized) structural tendencies (for a review see [62]). From this, parameter estimates can be obtained (using maximum likelihood procedures), which show the most probable node-level tendencies (e.g., to form links) that can produce the observed network. Models were generated using StOCNET (version 1.8). Networks were conditioned on density so that the parameter estimates were controlled for network size. Marcov Chain Monte Carlo Maximum Likelihood estimation procedures provided parameter estimates. None of the models degenerated, and parameter estimates successfully converged.

As predicted, networks differed in the micro tendencies of nodes to form links (more links implying higher density at the macro level). The strongly self-defining Christian network (B = -2.91, SE = 0.03; 95% CI: -2.84, -2.97) was significantly more dense (and thus more interconnected; *z* = 8.03, *p* < .00001; beta values compared using an approximate z distribution method) than the weakly self-defining network (B = -3.27, SE = 0.03; 95% CI: -3.20, -3.32). In contrast, there was no substantive difference between the strongly (B = -3.16, SE = 0.04; 95% CI: -3.08, -3.28) and weakly (B = -3.11, SE = 0.03; 95% CI: -3.05, -3.18) self-defining gender networks (*z* = 0.91, *p* >.18). (For a discussion of the negative betas see “first model” in S7 Appendix.)

In order to address the possible explanation that the higher density of the strongly self-defining religious network is due to high levels of interconnectedness within identities, not between them, a second model tested connectedness of overlapping identity-concepts with the rest of the network. For this set of models, density was entered as a control, in order to make sure that the observed tendency was not due to general density, and the tendency for overlapping identity-concepts to send ties was modelled as the key dependent variable. The tendency for overlapping identity-concepts within the strongly self-defining Christian network to be interconnected with all other nodes in the network was somewhat greater (B = 1.27, SE = 0.05; 95% CI: 1.18, 1.38) than the weakly self-defining network (B = 1.19, SE = 0.05; 95% CI: 1.09, 1.29), which approached significance (*z* = 1.26, *p* = .10; see “second model” notes in S7 Appendix). In sum, the strongly self-defining network was more interconnected than the weakly self-defining network, though this seems partly attributable to the observation that there is a greater proportion of overlapping nodes in the network as a whole. This higher connectedness was attributable to density across the whole network, including significantly more relations between overlapping identity-concepts and the rest of the network.

The tendency for overlapping identity concepts within the strongly (B = 1.27, SE = 0.05; 95% CI: 1.17, 1.37) and weakly (B = 1.24, SE = 0.05; 95% CI: 1.14, 1.34) self-defining gender networks to be connected to the rest of the network did not differ significantly from each-other (*z* = 0.38, *p* > .35). This confirms the visual inspection of networks above, and supports the conclusion that self-definingness of gender has no notable association with the fit between gender and religious identities.

#### Network analysis validity check

Importantly, network effects converged with self-reported measures. In order to test this, regressions were conducted with self-definingness of religious/gender identities as predictors, and the different inter-identity fit measures as outcome variables. Order of list presentation and age were controlled for, but neither was significant and they are therefore not reported. Firstly, self-definingness of Christianity was positively associated with personally experienced inter-identity content fit (B = 0.26, SE = 0.09, *t* (39) = 2.73, *p* < .01). Secondly, evidence of increased cognitive overlap between social identities as a function of religious self-definingness was found: The normalized number of overlapping words—repeated on both the participant’s female and Christian lists—was greater the more self-defining their religious identity (B = 0.002, SE = 0.001, *t* (39) = 2.14, *p* < .04). Finally, we conducted similar analyses with self-definingness of gender identity as the predictor. In contrast to religious identity, gender self-definingness did not predict any indices of inter-identity content fit significantly (*p*’s> .80). Thus, we conclude that both network generated and self-reported content measures confirm that, in contrast to self-defining religion, the self-definingness of gender identity has little discernible consequences for the fit between multiple social identities.

### Discussion

Study 3 offered a novel insight into the actual content of people’s Christian and gender identities through our application of a new measurement method. Specifically, we found that traits associated with these social identities were related to holisticness and self-definingness in the predicted way: Self-definingness of religiosity was associated with greater overlap between religious and gender identities and an increase in actual cognitive overlap. Thus, self-definingness of religion was associated with increased overlap between the actual social identity content of religion and gender. In line with our previous findings, self-definingness of gender was not associated with the integration of religious and gender identities. Identity content of highly self-defining religious participants, by contrast, comprised a web weaving together otherwise orthogonal facets of the self by increasing inter-identity fit. Thus, the Study 3 findings provide convergent evidence for our hypothesis and simultaneously validates our novel network method of tapping into inter-identity fit.

## General Discussion

Three studies using different methods documented converging evidence for our hypothesis that the inter-identity fit between multiple social identities varies according to group holisticness (i.e., the extent the group is consensually recognized to prescribe a philosophy for life; operationalized here as religious rather than gender identity) and self-definingness (the extent to which this philosophy is applied by the individual). For the less holistic based identity of gender, different aspects of identity were more independent of one another, regardless of its self-definingness. But for the more holistic based religious identity, a clear pattern of dependencies emerged. As predicted, only among strongly self-defining Christians, we found evidence of stronger inter-identity fit: The more self-defining one’s religious identity the greater the perceived fit, cognitive representations of inter-identity fit, and actual content-based overlap between it and the otherwise orthogonal, social identity of gender. These results were obtained consistently with three different measurement methods—explicit questionnaire responses (Studies 1), response latencies (Study 2) and a novel network analysis of identity content (Study 3). Below, we discuss the implications of our findings for theory, research, and practice.

### Theoretical implications

Our findings imply that it is important to consider both the type of group (i.e., group characteristic holisticness) and how they are personally applied (i.e., individual characteristic self-definingness). Results across all studies confirmed that only when a group was highly holistic, was stronger self-definingness related to stronger fit between Christian and gender identities. So, although holistic groups are “special” in the sense that they provide a philosophy for life and have the potential to promote inter-identity fit, this potential can only be realized by the individual’s application of group philosophies for life. It is ultimately individuals' self-definingness that allows these “special” group philosophies to strengthen inter-identity fit and come out, transcending different situations.

Of course, our results are not the first to emphasize the special role that social identities such as religion [14, 15, 63, 64] or political activism (e.g., ‘identity extension’, [65]; ‘personalism’, [66]) can play: These identities have a tendency to be all-defining in life. However, we move beyond this earlier work by concretely exploring what that means for the way such identities fit within the wider self-concept. Moreover, as far as we are aware, this research presents the first attempt to unify such varying identities under one rubric: Socially shared identity content (i.e., holisticness) provides the ideological basis for a life-philosophy, but only the individual’s use of that content (i.e., the self-definingness) ultimately actualises this potential (i.e., allows multiple social identities to fit together or overlap). Thus, our line of thought moves beyond previous conceptualizations of religious identity by viewing it as one (prototypical) member of a larger family of holistic groups.

The current work further offers a broad and nuanced perspective on the fit between multiple social identities. For instance, some have suggested that the fit between different facets of identity is neutral (i.e., independence) or negative [9], as are antagonistic inter-identity relations [19]. The present research suggests, in line with other work [2, 12, 35, 38, 40], that there is little support for universal antagonism and only qualified support for independence. But, perhaps more importantly, we also show that strong fit can emerge between multiple social identities in particular cases, such as those involving religion (as highly holistic and strongly self-defining) and gender. Notably, although this fit was strong at times, overlap between identities was never absolute (cf. mismatching ‘Match-Types’ in Study 2, and network idiosyncrasies in Study 3); identities did not become synonymous or merged into a unitary identity, but always retained some distinctiveness. In this way, the relation presented was between two unique identities, which shared important similarities and differences, but were shaped to be increasingly congruent with one-another. Thus, our line of thought suggests that the fit between multiple social identities can be thought of as reflecting a continuum ranging from non-overlapping to highly overlapping, varying as a function of both identity self-definingness and holisticness. Importantly, because gender is naturally occurring (i.e., not personally chosen) this observed increase in cognitive fit in strongly self-defining Christians should have been induced more organically through an internal process of increased inter-identity fit. Studies 2 and 3 indicate that holistic identities *transform* pre-existing social identity content, by mobilizing specific aspects of ancillary social identities that are consistent (or at least not inconsistent; e.g., loving) with its ideology, whilst diminishing the prominence of inconsistent traits (e.g., shopper). Holistic groups may therefore promote a more unified self-concept through the alignment of multiple, otherwise disparate, social identities. Together, our findings therefore suggest that the multifaceted self (e.g., [17, 21]) need not be inconsistent with individuals’ ability to achieve a more stable and unitary sense of self: Holisticness and self-definingness offer a pathway to achieving greater consistency over multiple and distinct self-aspects.

### Practical implications

Our findings also have more practical implications, particularly when considering the specific religious and gender identities involved in our studies. There is growing evidence in the literature to suggest the important role that a religious identity can play in the self-concept [63, 64, 67, 15]. The present findings provide support for this stance. Our results align with Allport and Ross’s [14] distinction between intrinsic and extrinsic religiosity, and provide the first empirical evidence concerning the intrapsychological consequences of intrinsic religiosity. Additionally, results converge with Ysseldyk et al. [63, 64], suggesting that the guiding, socially shared, belief-system offered by religions may be an important factor amplifying the cognitive and emotional importance of these identities, relative to other (less holistic) social identities. Finally, our results are also consistent with Koole et al. [15], who argue that religion can non-invasively influence all areas of an individual’s experience—including action, attention, affect, and perceived meaning—over diverse contexts, via implicit self-regulation. Our findings shed new light on what factors may underlie those arguments: not just individual-level self-definition, but also socially shared identity content.

Regarding gender, our research clearly shows it to be a highly influential identity for many in our sample. But despite this, gender does not define inter-identity fit in the same way that religion does. We attribute this to the lack of a uniform ideology attached to gender identity content: Although there is nothing to stop gender from regulating relations among different aspects of self, in the general population based samples we recruited, it was not clearly related to inter-identity fit. Importantly however, we expect that in particular contexts (e.g., politicization), gender identity can transform, taking on a new personal meaning (e.g., feminism) and thereby come to define the whole self—and influence inter-identity fit—in ways that are more consistent across the whole category [68].

### Limitations and directions for future research

Although Study 3 employed a semantic network analysis to test our hypothesis, one may wonder about its validity. Indeed, although the methods of social network analysis used in our approach are tried and trusted [69, 61], the application to self-reported identity-associations is entirely novel, as far as we know. As with all new methods, the validity of this method needs thorough assessment. Nevertheless, the results of this study converged with the other measurement methods we used. More generally, we are quite optimistic about what this method has to offer. For instance, it is very easy to administer, and can be administered to measure identity content of any social group or to any aspect of identity. Furthermore, this method is relatively implicit and non-reactive, and allows for both description and quantification. So, whereas the RT task in Study 2 might be criticised for the pre-selection of traits (which may suffer from confounded meaning). ART does not suffer from such limitations because choice and meaning were obtained from the participants themselves. Future research should embrace this new method to test its potential to measure identity content.

One valuable application of the ART method could be in addressing the juncture between holisticness/self-definingness and the intersectionality literature [35, 36]. Both research areas converge on their aim to explore the fit between multiple identities, but holisticness/self-definingness focuses more strongly on why this occurs, while intersectionality tends to focus on the how meaning of these identities depends on each other [35]. Thus, in many ways, these theories are complementary. Moreover, considering the aspect of meaning creation between multiple identities, we agree that intersections between identities can be important cognitive spaces for transformation of identity content (e.g., using a group philosophy to increase overlap between identities). Although the extent to which the fit highlighted in the present paper are additive or transformative remains an empirical question, ART makes it possible to distinguish these types of relations by comparing (1) the intersectional female-Christian identity, with (2) the intersection between the two social categories of female and Christian. This method may therefore be useful for empirically supplementing and corroborating claims in the intersectionality literature [70].

We further note that our choice of samples and groups necessarily limits the generalizability of our findings. Firstly, although we recruited diverse samp7les (student and community samples from the Netherlands and U.S., respectively), they were all taken from individualist societies. Conclusions may therefore be limited to independent cultures. Nevertheless, the findings by Baray et al. [41] among Turkish national action party members suggest comparable processes may also be found in holistic groups in non-Western cultures too. Secondly, the inclusion of non-religious individuals in our sample means that results should be interpreted with caution. Importantly however, we had theoretical reasons for including these individuals, and no substantive statistical differences were found between religious and non-religious individuals (see S5 Appendix). Thirdly, it may be the case that self-selection (into one’s religious group, but not one’s gender group) may have biased our findings. Although including non-religious people in our sample arguably helps to alleviate this critique, possible alternative explanations concerning self-selection of high identifiers of course remain; for example it could be the case that those who are devotedly religious have a need for cognitive structure and simplicity (cf. [37]). Importantly however, we believe that self-selection does not necessarily question the presence of the relations reported, because inter-identity fit (i.e., between religion and gender) were measured within the same participants: Inter-identity fit is not an individual difference characteristic based on personal predispositions, but concerns the way that individuals apply holistic ideologies.

A potentially more pressing limitation of the present research is that it only focuses on Christianity and gender. We made this deliberate choice on in order to ‘cleanly’ compare three studies employing different methods to tap into inter-identity fit. Thus, we present strong evidence supporting the stronger fit between gender and religion when religion is strongly self-defining. However conclusions drawn from these analyses are, for now necessarily limited to these groups. Nevertheless, our findings equally provide some preliminary support for the utility of these constructs and their potential to apply more widely: The Pilot study hinted towards wider applications to different groups (including environmental, health and nationality) whereas similar processes to those highlighted in the present research also emerged in Extremist Turkish political organisations [41], as in Muslim religious identity and martial arts groups who prescribed a particular philosophy [43]. Finally, we are also currently in the process of gathering and analysing data for a promising replication of the present effects using the environmental identity.

One more specific concern regarding generalizability is that Christianity may simply have exerted a direct influence on gender identity content, and that this confounded our results. Indeed, the Bible explicitly informs gender roles (e.g., 1 Timothy 2: 9–15, New International Version). Although further tests in other religions with less clear doctrines (e.g. humanism) may help to address this limitation in the future, we also have some evidence that alleviates this concern. Specifically, we find variance in the extent that religious identities are found to define gender identities—which varies in association with self-definingness. Moreover, there was some suggestion that stronger inter-identity fit was facilitated through a bidirectional process (i.e., gender and religion mutually informing each other; e.g., Study 3 female words such as ‘strong’ becoming associated with religion in strongly self-defining women only, and in Study 1 self-definingness of religion was associated with the fit of religion in gender and vice versa), rather than a simple relation of gender being defined by religion. Indeed, Study 3 showed that overlap between religion and gender was achieved on content which may not be archetypally religiously defined (i.e., strong, focused).

By and large, we are optimistic that our findings do not reflect a quirky exception due to Christianity alone. Future research can build on the present work by corroborating its external validity and addressing its internal validity. As for external validity, future research should focus on and compare different religions and aspects of fit between Christianity and gender, for example, under conditions where fit may come more strongly under question (e.g., for homosexual Christians’ gender or sexual identity; [11]. Furthermore, we think it is important to explore beyond religious identities to other holistic identities, such as those of politicized activists or professional athletes. This could give us insight into how fit between multiple identities within activists or athletes can aid their pursuit of a philosophy for life in the face of potentially competing goals (e.g., goal of lowing carbon footprint v. goal to visit foreign and distant country). As for internal validity, future research could manipulate group holisticness (i.e., by manipulating the extent a group is perceived to prescribe a philosophy for life). However, it is unclear how viable it would be to test the consequences of holisticness experimentally: Given that inter-identity fit is an outcome of both holisticness and self-definingness it is unclear whether intrapersonal consequences (i.e., varying inter-identity fit) will be observable in short experimentally controllable periods. Despite this, gender may be an ideal identity to utilise for such a procedure, because results demonstrate that (a) it does not generally function as highly holistic groups do, and (b) individuals accept it can be strongly self-defining.

## Conclusion

The modern Western human is often faced with the challenge of juggling multiple identities, which may be potentially independent, or even in outright conflict with each other. Our findings converged across three studies to suggest that the holistic group of religion may resolve such identity complexities by promoting a more harmonious fit between potentially conflicting identities (such as gender). Importantly, however, our findings indicate that it is not religion itself (or its philosophies) that synthesize the fragmented self, but the individual’s application of the group’s philosophy for life which gives their different roles and identities a sense of place.

## Supporting Information

S1 Appendix The theoretical and methodological distinction between self-definingness and identification. This appendix outlines the theoretical distinction between identification and self-definingness, discusses Pilot bivariate correlations between identification and self-definingness and reports a factor analysis of identification and self-definingness.
(DOCX)

S2 Appendix Hypothesis notes. A more detailed discussion about expectations concerning groups low in holisticness.
(DOCX)

S3 Appendix Holisticness, Self-definingness and inter-identity fit items. (DOCX)

S4 Appendix Factor analysis of holisticness and self-definingness. (DOCX)

S5 Appendix Sample composition. Discussing the inclusion of non-religious individuals in the sample.
(DOCX)

S6 Appendix Study 2 methodology notes. Discussing the inclusion of the self in the RT task.
(DOCX)

S7 Appendix Semantic Networks Analysis: Methodological Notes. Appendix including additional notes on Study 3 method and results. Method notes discuss the use of a median split in network construction, the window size used to code ties (relations) between nodes (identity concepts), and notes on data cleaning and coding. Results notes discuss the Figures, negative density betas in the first model, and marginal results in the second model.
(DOCX)
