# Supplementary material for: Achieving Harmony among Different Social Identities within the Self-Concept: The Consequences of Internalising a Group-Based Philosophy of Life
Source: PLoS One. 2015 Nov 30;10(11):e0137879. doi: 10.1371/journal.pone.0137879 (PMC4664279; doi:10.1371/journal.pone.0137879)
Supplement: S1 Appendix — This appendix outlines the theoretical distinction between identification and self-definingness, discusses Pilot bivariate correlations between identification and self-definingness and reports a factor analysis of identification and self-definingness. (DOCX) [file pone.0137879.s001.docx]

**S1 Appendix**

**The theoretical and methodological distinction between self-definingness and identification**

**The theoretical distinction between self-definingness and identification**

It is the context independent application of group philosophies which characterizes self-definingness and crucially differentiates it from other aspects of collective identity such as identification [71] and self-categorization [21]. Firstly, self-definingness focuses on a specific element of group content (i.e., an individual’s application of group philosophies), while identification focuses on a broader aspect of one’s general relationship with the group in general. Secondly, self-definingness focuses on how an individual applies these group philosophies, to their whole life (i.e., independent of context), while both identification and self-categorization are context dependent. For example, an individual may strongly identify strongly with fans of a t.v. show, but this identity may only inform this individual’s behaviour in particular social settings (when watching the program) and not others (when eating soup)–the identity is consequently weakly self-defining. Conversely, a very religious individual may consider religion to be so strongly self-defining so that it guides their actions and thoughts in almost all social and personal settings. Thus, neither identification (which taps into one’s relationship to a group) nor self-categorization (which measures one’s context dependent classification of self as a group member), is sensitive to the degree to which a social identity informs the entire self or merely a significant part of it in the same way as self-definingness.

**The methodological distinction between self-definingness and identification**

**Pilot bivariate correlations exploring identification and self-definingness.** Pilot results highlight some clear relations between identification and self-definingness. For example, Pilot study correlations between identity fit and self-definingness/identification are highly comparable. This aligns with the factor analysis reported below which indicate that self-definingness fits as a separate factor correlated with self-investment and self-definition components of identification. Importantly however, self-definingness is not synonymous with identification theoretically or empirically. Pilot study correlations between self-definingness and identification are also moderated by holisticness in theoretically consistent ways. Correlations were high (and almost interchangeable) for highly holistic identities: For identities which prescribe a life philosophy identifying with a group is indeed synonymous with self-definingness, in that high identifiers will internalize and apply the group’s philosophies to life in general in the same way as high self-definers do. But for less holistic groups, that do not prescribe a clear philosophy for life, identifying with this group does not necessarily mean that the individual applies the group’s philosophies to life in general, instead individuals may only apply the group to specific contexts. In this case, identification may be high and self-definingness may be low. This indicates that although identification and self-definingness share some expected commonalities, if we want to explore the consequences of applying a philosophy for life, then it is valuable to focus on self-definingness.

**Factor analysis of identification and self-definingness.** Using the data collected in Study 1, a factor analysis was conducted to explore the latent structure of the self-definingness scale in relation to the 14-item [72] identification scale. Analyses were conducted using the Lavaan (version 0.5.10) package in R (version 12.5.1). Models were estimated using Maximum Likelihood estimation with robust (Huber-White) standard errors [73]. Absolute fit indices of CFI, TFI and SRMR were reported, along with indices of parsimony, RMSEA, and comparative fit, AIC. Guidelines suggest that CFI and TFI should be greater than .95, SRMR should be close to .08 or below, and RMSEA should be .06 or below [74]; [75]. Results are presented in Table A.

We began by conducting a confirmatory factor analysis which explored the seven self-definingness items regarding, first Christian, and second, the female identity. We expected that all seven-items would load onto a single latent factor. This model was a good fit for gender ($\mathcal{X}^{2}$ (14) = 18.22, *p* < .20), and acceptable fit for the Christian data ($\mathcal{X}^{2}$ (14) = 30.61, *p* < .007). An inspection of standardized factor loadings (Range $\beta$ = .82 - .95) suggested high levels of variance in each item were explained by a single latent factor (Range = 67% - 91%). A further inspection of modification indices suggested that the items 1 and 2 shared unexplained covariance and should be correlated. This addition to the model resulted in a significant improvement in model fit ($\mathcal{X}^{2}$ difference (1) = 11.90, *p* < .001), and a very good overall model, in terms of absolute fit, parsimony and comparative fit. Thus, the one-factor solution we expected was confirmed by the data.

Next, we explored the relation between self-definingness and identification [72]. We expected that self-definingness would be a separate factor from the two dimensions of identification in [72]: self-investment (a dimension with 3 components: satisfaction, solidarity and centrality) and self-definition (with 2 components: homogeneity and self-stereotyping). Additionally we assumed that self-definingness would be conceptually most strongly related to self-investment (cf. [49]). Three models were run for Christian and gender variables, respectively. Model 1 specified self-definingness as a separate factor correlated with self-investment and self-definition, in the hierarchical structure specified by [72]. Model 2 specified self-definingness as a 4^th^ component of self-investment, and Model 3 specified self-definingness as 3^rd^ component of self-definition.

Results for Christianity confirmed hypotheses. Model 1 was significantly better than Model 2 and 3 in terms of absolute fit to data. Indeed, the three separate factor solution leads to a significant reduction in Chi-Square ($\mathcal{X}^{2}$ _Model 1 v. 2 difference_ (1) = 5.72, *p* < .05; $\mathcal{X}^{2}$_Model 1 v. 3 difference_ (1) = 11.22, *p* < .001), and a lower AIC. Additionally, our expectation that self-definingness would be more strongly related to the self-investment factor than self-definition was confirmed by factor covariances (COV _self-definingness: self-investment_ = 1.53 (SE = .27); COV _self-definingness: self-definition_ =.05 (SE = .25)). Moreover, a comparison of Chi-Squared differences derived from the baseline model suggested that Model 2 was a slightly better fit than Model 3.

The results for gender are quite consistent with predictions, too. There is very little distinction between Models 1 and 2: they have almost identical fit indices. It is therefore possible to consider self-definingness as a separate factor, or alternatively as a component of self-investment. What is clear, however, is that both Model 1 and 2 have a better fit than Model 3.

Taking both sets of analyses together, our factor analysis confirmed that self-definingness is a single factor, which is related to other dimensions of identification, but nonetheless distinct (especially so for religion). Thus, the factor analysis provides some evidence for convergent and divergent validity of the self-definingness construct.

**References**

1. Ashmore RD, Deaux K, McLaughlin-Volpe T. An organizing framework for collective identity: articulation and significance of multidimensionality. Psychol Bull. 2004 Jan; 130: 80-114.
2. Leach, CW, van Zomeren M, Zebel S, Vliek M, Pennekamp SF, Doosje B et al. Group-level self-definition and self-investment: A hierarchical (multicomponent) model of in-group identification. J Pers Soc Psychol. 2008;95: 144-165.
3. Rosseel Y. Lavaan: An R Package for structural equation modeling. J Stat Softw. 2012;48: 1-36.

Table A. Confirmatory factor analysis goodness-of-fit indicators testing self-definingness, identification and a combined model for Religious Identity and Gender Identity.

| Model | | $\mathcal{X}^{2}$ | df | $\mathcal{X}^{2}$ difference, df | CFI | TLI | RMSEA | (90% CI) | SRMR | AIC |
| --- | --- | --- | --- | --- | --- | --- | --- | --- | --- | --- |
| Religious Identity Models | |  |  |  |  |  |  |  |  |  |
|  | Self-definingness (SD) | 30.61* | 14 | 538.16, 7 | 0.97 | 0.95 | 0.09 | 0.06 - 0.13 | 0.02 | 2,657.24 |
|  | SD with covariance correlation | 19.52 | 13 | 11.90, 1 | 0.99 | 0.98 | 0.06 | 0.00 - 0.10 | 0.01 | 2,638.83 |
|  | Leach | 86.22 | 71 | 1289.05, 20 | 0.99 | 0.99 | 0.04 | 0.00 - 0.06 | 0.03 | 5,411.47 |
|  | Model 1 | 294.74** | 181 | 2288.97, 29 | 0.95 | 0.94 | 0.07 | 0.06 - 0.08 | 0.05 | 7,949.17 |
|  | Model 2 | 300.45** | 182 | 2283.25, 28 | 0.95 | 0.94 | 0.07 | 0.06 - 0.08 | 0.05 | 7,955.64 |
|  | Model 3 | 305.96** | 182 | 2277.75, 28 | 0.95 | 0.94 | 0.07 | 0.06 - 0.08 | 0.05 | 7,962.31 |
| Gender Identity Models | |  |  |  |  |  |  |  |  |  |
|  | Self-definingness | 182 | 14 | 200.89, 7 | 0.98 | 0.97 | 0.05 | 0.00 - 0.10 | 0.04 | 2,909.61 |
|  | Leach | 85.82 | 71 | 715.69, 20 | 0.98 | 0.97 | 0.04 | 0.00 - 0.07 | 0.05 | 4,873.28 |
|  | Model 1 | 242.72* | 181 | 999.85, 29 | 0.94 | 0.93 | 0.05 | 0.03 - 0.07 | 0.07 | 7,750.73 |
|  | Model 2 | 242.78* | 182 | 999.79, 28 | 0.94 | 0.93 | 0.06 | 0.03 - 0.07 | 0.07 | 7,749.25 |
|  | Model 3 | 250.80* | 182 | 99.177, 28 | 0.93 | 0.92 | 0.05 | 0.04 – 0.07 | 0.07 | 7,756.56 |

Note. * *p* <.05, ** *p* < .001

All $\mathcal{X}$^2^ difference is relative to a baseline model, which assumes no covariances between the observed variables, with the exception of the Religious Identity Model ‘Self-definingness, with covariance correlation’, which is measured relative to the ‘Self-definingness’ Model it is nested in.
