# Supplementary material for: Achieving Harmony among Different Social Identities within the Self-Concept: The Consequences of Internalising a Group-Based Philosophy of Life
Source: PLoS One. 2015 Nov 30;10(11):e0137879. doi: 10.1371/journal.pone.0137879 (PMC4664279; doi:10.1371/journal.pone.0137879)
Supplement: S2 Appendix — A more detailed discussion about expectations concerning groups low in holisticness. (DOCX) [file pone.0137879.s002.docx]

**S2 Appendix**

**Hypothesis Notes**

Although we theoretically expect that a group low in holisticness can become strongly self-defining (e.g., a Star Trek viewer who becomes a ‘Trekkie’), we expect that this occurrence will be relatively rare. Thus, when we obtain a sample from the general population, self-definingness of groups low in holisticness will not influence inter-identity fit. Consequently, our hypothesis is based on the assumption that we are testing these effects in a general population sample. However, if we were to gain our sample from a specific, more limited population (e.g., attendendee’s of a feminist convention) then we would expect self-definingness of gender to influence inter-identity fit.
