# Supplementary material for: Achieving Harmony among Different Social Identities within the Self-Concept: The Consequences of Internalising a Group-Based Philosophy of Life
Source: PLoS One. 2015 Nov 30;10(11):e0137879. doi: 10.1371/journal.pone.0137879 (PMC4664279; doi:10.1371/journal.pone.0137879)
Supplement: S3 Appendix — (DOCX) [file pone.0137879.s003.docx]

**S3 Appendix.**

**Holisticness, Self-definingness and inter-identity fit items**

**Holisticness of Christianity [Gender]**

Christianity [Gender] gives people a foundation to build their lives upon.

Christianity [Gender] promotes a specific set of ideals.

Christianity [Gender] gives guidelines for what is good or bad behaviour.

Christianity [Gender] provides a philosophy for life.

Christianity [Gender] teaches people how to live.

**Self-definingness of Christianity** [Gender]**.**

My everyday decisions are influenced by Christian [female] values.

My personal goals are informed by Christian [female] principles.

Religion [Gender] is irrelevant to how I live.^reversed^

Christianity [Being female] helps me to see what is important in life.

Being Christian [female] gives me a sense of purpose.

My religion [gender] doesn't say a lot about the real me.^reversed^

Being Christian [female] is an important part of who I am.

Christianity [Womanhood] steers me through life.

Other factors in my life have a much stronger influence on me than my religion [gender].^reversed^

Christianity [Womanhood] informs my everyday decisions and actions.

**Inter-identity fit of Christian in female [female in Christian].**

Being a Christian [woman] is consistent with being a woman [Christian].

The principles valued by Christians [women] cannot be rhymed with the principles valued by women [Christians].

The values of Christians [women] are compatible with female [Christian] values.

The behaviours accepted by Christians [women] are also accepted by women [Christians].

Characteristics typical of Christians [women] are similar to those associated with being a woman [Christian].
