# Supplementary material for: Achieving Harmony among Different Social Identities within the Self-Concept: The Consequences of Internalising a Group-Based Philosophy of Life
Source: PLoS One. 2015 Nov 30;10(11):e0137879. doi: 10.1371/journal.pone.0137879 (PMC4664279; doi:10.1371/journal.pone.0137879)
Supplement: S4 Appendix — (DOCX) [file pone.0137879.s004.docx]

**S4 Appendix**

**Factor analysis of holisticness and self-definingness**

A series of factor analyses were conducted to check the distinction between holisticness and self-definingness scales within each of the five groups tested: Two models (per group) were run to test this relation; Model 1 specified self-definingness as a separate factor correlated with holisticness, Model 2 specified a single factor with which all self-definingness and holisticness items were related. Models were estimated using Maximum Likelihood estimation with robust (Huber-White) standard errors [73]. Absolute fit indices of CFI, TLI and SRMR were reported, along with indices of parsimony, RMSEA, and comparative fit, AIC. Guidelines suggest that CFI and TLI should be greater than .95, SRMR should be close to .08 or below, and RMSEA should be .06 or below [74]; [75]. Analyses revealed that Model 1 always had a better fit (Chi Square: 3.23 -11.78, *p* = .52 - .001; CFI: .94-1.00; TLI: .86-.95, SRMR: .01 - .04) and parsimony (Model 1 RMSEA: .00-.17) than Model 2 (Chi Square: 11.87 - 82.85, *p* = .01 - .00001; CFI: .70-.94; TLI: .40-.88, SRMR: .04-.13; RMSEA: .11-.37). Moreover the AIC for comparative fit was always lower for Model 1 than 2 (AIC_difference_: 3.57– 150.83). This suggests that although the one factor solution may be viable –in terms of a decent fit to the data – theoretical and methodological grounds support the separation of these items into two holisticness and self-definingness factors.

**References**

1. Rosseel Y. Lavaan: An R Package for structural equation modeling. J Stat Softw. 2012;48: 1-36.
2. Hu L, Bentler PM. Cutoff criteria for fit indexes in covariance structure analysis: Conventional criteria versus new alternatives. Struct Equ Modeling. 1999;6: 1–55.
3. Brown TA. (2006). Confirmatory factor analysis for applied research. 1st ed. New York: Guilford Publications; 2004.
