# Supplementary material for: Achieving Harmony among Different Social Identities within the Self-Concept: The Consequences of Internalising a Group-Based Philosophy of Life
Source: PLoS One. 2015 Nov 30;10(11):e0137879. doi: 10.1371/journal.pone.0137879 (PMC4664279; doi:10.1371/journal.pone.0137879)
Supplement: S5 Appendix — Discussing the inclusion of non-religious individuals in the sample. (DOCX) [file pone.0137879.s005.docx]

**S5 Appendix**

**Sample composition**

We included non-religious individuals in the sample because theoretically we are interested in how individuals use Christian group philosophies rather than the category membership. Because actively identifying with religion in modern society is often considered a mark of deep conviction [76] it is important to also test these individuals with a comparison group for whom this identity is less self-defining or even not self-defining. We therefore conducted our studies in countries with a Christian heritage, expecting similar processes to function in both religious and non-religious people legitimizing their pooling in our sample. We argue that, especially in this case, self-categorization is not necessary for an individual to apply group ideologies, in that non-religious individuals are capable of tapping into the meaning of the group ideologies, but do not seek to apply them as actively to their lives as those who are strongly self-defining. In order to test this notion statistically, the regression analyses presented in Study 1, 2 and 3 were re-run excluding non-religious individuals. Effects replicated with a few exceptions. For study 1, there were no substantive differences except for inter-identity fit: The self-definingness of women became predictive of both female in Christian fit (B=0.30, SE =0.11, *t* (61)=2.70, *p* <.008) and vice versa (B=0.24, SE =0.11, *t* (61)=2.12, *p* <.04), while the self-definingness of religion predicted fit of Christian in female (B=0.17, SE =0.16, *t* (61)=2.69, *p* <.008), but was marginal for the fit of female in Christians (B=0.12, SE =0.07, *t* (61)=1.78, *p* =.08). Importantly regression among the non-religious sample were as expected: Self-definingness of religion explained the fit of religion in female identity (B=0.40, SE=0.13, *t* (65) =3.23, *p* <.003) and marginally vice versa (B=0.24, SE=0.13, *t* (65) =1.91, *p =*.06), but the self-definingness of women did not predict either (*p*’s >.59). Similarly, testing Study 2 in the religiously categorizing sample replicated the crucial hypothesis testing interaction effect, with self-definingness of religion being associated with speeded responses to traits matching the self, religion and gender (B=-0.025, SE = 0.013, *z* = 1.92 , *p* <.03), while no effects of self-defining gender were found (p’s >.14). However, in this case we did not find evidence of speeded effects of a match between self and religion according to the self-definingness of religion (*p* =.08). Thus, as could be expected, analyses show that our predicted pattern of findings applies to all participants tested, though most strongly to those who are religious. More importantly, findings emphasize that also for non-religious people in a Western context, Christianity is not separate from the self-concept, but influences inter-identity fit in the expected way. This provides some statistical justification for pooling these samples, enabling us to look beyond the ‘special case of religion’ to the application of group philosophies in general.

**References**

1. Taylor C. A secular age. 1st ed. Cambridge: Belknap Press of Harvard University Press; 2007.
