# Supplementary material for: Achieving Harmony among Different Social Identities within the Self-Concept: The Consequences of Internalising a Group-Based Philosophy of Life
Source: PLoS One. 2015 Nov 30;10(11):e0137879. doi: 10.1371/journal.pone.0137879 (PMC4664279; doi:10.1371/journal.pone.0137879)
Supplement: S6 Appendix — Discussing the inclusion of the self in the RT task. (DOCX) [file pone.0137879.s006.docx]

**S6 Appendix**

**Study 2 methodology notes**

Study 2’s methodology was chosen to replicate and extend the methodology of [45] by including an additional social identity in their original paradigm, which measured the relation between the self and one social identity. Their research suggests that excluding the self from this analysis would result in substantial unexplained variance considering the measurement of the relation between religion and gender contingent upon whether the matches between religion and gender mismatched the self or not; an assertion supported by our own analyses, as indicated by the significant difference between self-identities mismatch and self-identities match beta contrasts presented in Table 3. Thus, we believe that this step-wise progression in the Smith-Henry paradigm allows more accurate exploration of our hypothesis and most effective comparison with prior research.
