# Supplementary material for: Achieving Harmony among Different Social Identities within the Self-Concept: The Consequences of Internalising a Group-Based Philosophy of Life
Source: PLoS One. 2015 Nov 30;10(11):e0137879. doi: 10.1371/journal.pone.0137879 (PMC4664279; doi:10.1371/journal.pone.0137879)
Supplement: S7 Appendix — Appendix including additional notes on Study 3 method and results. Method notes discuss the use of a median split in network construction, the window size used to code ties (relations) between nodes (identity concepts), and notes on data cleaning and coding. Results notes discuss the Figures, negative density betas in the first model, and marginal results in the second model. (DOCX) [file pone.0137879.s007.docx]

**S7 Appendix**

**Semantic Networks Analysis: Methodological Notes**

**Method**

**Using a median split to construct the networks.** A median split was necessary for this analysis on methodological grounds. Firstly, it is not possible to analyse networks as a continuous variable. Secondly, network descriptives (e.g., density) are very sensitive to network size, thus using the median increases comparability of network descriptives across networks. Importantly, because of the low correlation between self-definingness of religion and gender (*r* = .01), these factors worked out as being quite orthogonal. The few participants who were on the median were allocated so that balanced composition of groups was ensured. For example, the weakly self-defining religious group (N= 21) consisted of 9 participants with weakly self-defining gender identity, and 12 for whom gender was strongly self-defining. The strongly self-defining group (N = 22) consisted of 12 with weakly self-defining gender and 10 strongly self-defining gender participants.

**Window Method***.* This window of four (i.e., the concept itself and three adjacent concepts) codes for a bi-directional relation between concepts recalled proximately on participants list—assuming these concepts to be more closely associated than those outside the window. Preliminary analyses were run with different window sizes (two, four and six), and no substantive differences emerged. We preferred the window of four because it matches within-list relations optimally given the range of identity concepts listed (3-20, *M* = 10.81, *SD* = 5.18), avoiding a loss of information resulting from the over-inflation or underestimation of within list relations associated with the more extreme window sizes.

**Associative Recall Data Cleaning.** Table A outlines cleaning of associative recall data, showing the original and generalized word.

**Table A. Original words listed in Study 3 networks and their cleaned versions.**

| Original word | Generalized word | Original word | Generalized word |
| --- | --- | --- | --- |
| selfless | Altruistic | varied | diverse |
| self-sacrificing | Altruistic | big on converting people | evangelists |
| sacrificing | Altruistic | womanly | feminine |
| great | Awesome | some of my friends | friends |
| attractive | Beautiful | tender | gentle |
| believers in god | Believers | mild | gentle |
| believing | Believers | gossips | gossipy |
| courageous | Brave | hard workers | hardworking |
| able | Capable | homemakers | housekeepers |
| caretakers | Caregivers | people | human |
| caring | Caring | often hypocritical | hypocritical |
| concerned for others | Caring | hypocrite | hypocritical |
| life givers | child-bearers | Jesus lovers | Jesus loving |
| church | Churchgoing | Joyous | joyful |
| many | Common | judging | judgmental |
| Numerous | Common | likeable | likable |
| worldwide | Common | lovely | loveable |
| community involved | Communal | hormonal | moody |
| community-minded | Communal | filled with integrity | moral |
| group-oriented | Communal | normal people | normal |
| neighborly | Communal | optimist | optimistic |
| Have compassion | Compassionate | girlfriend | partner |
| compasive | Compassionate | a force to be reckoned with | powerful |
| musical | Creative | prayers | prayerful |
| artistic | Creative | pure hearted | pure |
| clingy | Dependent | dependable | reliable |
| devout | Devoted | sensitive | sensitive |
| devout | Devoted | petite | small |
| industrious | Diligent | sociable | social |
| discriminated against | Discriminated | talkers | talkative |
| oppressed | Discriminated | not always big fans of science | unscientific |
| not respected | Discriminated | zealots at times | zealots |
| under paid | discriminated |  |  |
|  |  |  |  |

**Overlap coding***.* To increase robustness of the findings in Tables 5 and 6, traits were only coded as overlapping when the overlap was due to repetition on more than one person’s list. Thus, there was some degree of consensus about what traits were overlapping and unique.

**Results**

**Figure notes**. The network visualization software (ORA, version 2.2.9) applies a standard ‘spring embedder algorithm’ to define the layout of the networks [77]. This algorithm is based on the premise that nodes want to be as far away from each other as possible, and are pulled together only by the force of links. Thus, the proximity of the nodes in the visualization reflects the levels of connectedness in the network.

**First Model: Testing micro tendencies of nodes to form links**. The density parameters are negative because the frequency of links is compared to a mathematical standard of interconnectedness which is high compared to the expected interconnectedness when using the trait list generation method of this study.

**Second Model: Testing connectedness of overlapping identity-concepts with the rest of the network.** We believe that the trend for overlapping identity-concepts within the strongly self-defining Christian network to be marginally significantly more interconnected with other nodes in the network (than in the weakly self-defining network), disguises a quite strong difference, but that the analytic method lets us down. ERG models are not yet capable of dealing with weighted networks: links that occurred more frequently within the network (e.g., strong to hard-working link, N = 7) are weighed equally to ties occurring only once. But it would make sense for the former traits, which are consensually linked to other identity concepts, to be particularly interconnected in the strongly self-defining network. In order to test this we conducted the same analysis again, now considering only links that were identified by *two or more participants.* In this more robust analysis, there are significantly more interconnections in the strongly self-defining network (B= 1.75, SE= 0.11; 95% CI: 1.55, 1.96) than in the weakly self-defining network (B= 1.18, SE= 0.05); 95% CI: 1.02, 1.29), *z* = 4.80, *p* <.00001. By contrast, the strongly (B= 1.51, SE= 0.10; 95% CI: 1.32, 1.70) and weakly (B= 1.65, SE= 0.10; 95% CI: 1.47, 1.84) self-defining gender networks do not differ (*z* = 1.03, *p* = .15).

**References**

1. Carley K, Reminga J, Storrick J, Columbus D. ORA User's Guide 2010; 2010. Carnegie Mellon University, School of Computer Science, Institute for Software Research, Technical Report, CMU-ISR-10-120. Accessed 20 May 2014.
